# Diurnal cycles drive rhythmic physiology and promote survival in facultative phototrophic bacteria

**DOI:** 10.1038/s43705-023-00334-5

**Published:** 2023-11-24

**Authors:** Camille Tinguely, Mélanie Paulméry, Céline Terrettaz, Diego Gonzalez

**Affiliations:** Laboratory of Microbiology, Institute of Biology, University of Neuchâtel, Neuchâtel, Switzerland

**Keywords:** Water microbiology, Bacterial genetics

## Abstract

Bacteria have evolved many strategies to spare energy when nutrients become scarce. One widespread such strategy is facultative phototrophy, which helps heterotrophs supplement their energy supply using light. Our knowledge of the impact that such behaviors have on bacterial fitness and physiology is, however, still limited. Here, we study how a representative of the genus *Porphyrobacter*, in which aerobic anoxygenic phototrophy is ancestral, responds to different light regimes under nutrient limitation. We show that bacterial survival in stationary phase relies on functional reaction centers and varies depending on the light regime. Under dark-light alternance, our bacterial model presents a diphasic life history dependent on phototrophy: during dark phases, the cells inhibit DNA replication and part of the population lyses and releases nutrients, while subsequent light phases allow for the recovery and renewed growth of the surviving cells. We correlate these cyclic variations with a pervasive pattern of rhythmic transcription which reflects global changes in diurnal metabolic activity. Finally, we demonstrate that, compared to either a phototrophy mutant or a bacteriochlorophyll *a* overproducer, the wild type strain is better adapted to natural environments, where regular dark-light cycles are interspersed with additional accidental dark episodes. Overall, our results highlight the importance of light-induced biological rhythms in a new model of aerobic anoxygenic phototroph representative of an ecologically important group of environmental bacteria.

## Introduction

In natural environments, free-living bacteria face, between short intervals of fast multiplication, long periods of stasis or very slow growth [1–3]. To survive such periods, they have evolved diverse strategies to optimize resource utilization and spare energy [4–7]. Facultative photoheterotrophy is such a strategy that a subset of environmental bacteria use to supplement their mostly heterotrophic growth. It can be based on two main molecular mechanisms: retinalophototrophy, which uses rhodopsins to pump ions through the membrane, and chlorophototrophy, which is based on photochemical reaction centers containing chlorophylls or bacteriochlorophylls [8]. Aerobic Anoxygenic Phototrophy, hereafter AAnP, is a common chlorophototrophic pathway, whereby usable energy is produced from light under aerobic conditions in the absence of H_2_O dissociation and O_2_ release [9, 10].

Aerobic anoxygenic phototrophic bacteria include, among others, members of the alpha-, beta- and gammaproteobacteria [11]. These represent an important fraction of the prokaryotes present in the upper layers of seas and lakes, but are also widespread in environments as diverse as the phyllosphere, soil crusts, or thermal springs [12–14]. These bacteria encode a set of about 45 genes required to produce bacteriochlorophyll *a*, the main pigment needed for phototrophy, and the components of their light-harvesting complexes, including carotenoids [15]. While these bacteria are in general not able to grow photoautotrophically, with the notable exception of *Dinoroseobacter shibae* which can use the ethylmalonyl-CoA pathway [16], they can benefit from light exposure to maintain higher concentrations of intracellular ATP, incorporate limited amounts of CO_2_ via substrate co-assimilation or anaplerotic reactions, and spare organic carbon through downregulation of respiration [17–21]. However, bacteriochlorophyll *a* biosynthesis and AAnP represent a significant energy investment and are known to generate toxic byproducts, especially singlet oxygen and other reactive oxygen species, in the presence of oxygen and light [11, 22, 23]. That is why many aerobic anoxygenic phototrophs tend to regulate bacteriochlorophyll *a* production strictly and limit its synthesis to dark phases [24, 25].

The life history of aerobic anoxygenic phototrophs is likely to be deeply affected by diel cycles, both because these bacteria can actively utilize light when needed and because they tend to associate and cooperate with obligate photoautotrophs [23, 26–29]. While we know the preferences of some species in terms of light intensity and adaptations to non-optimal light conditions [30, 31], our understanding of the rhythmic physiology of these bacteria in response to diurnal cycles and more generally of the adaptive value of AAnP under different light regimes is still limited [32]. Indications can be found scattered in literature pertaining to quite diverse microbiological fields. Field studies in microbial ecology suggest that the transcription of AAnP genes may vary throughout the day, being maximal at night, and that cell division and cell activity are also driven by diurnal cycles in AAnP-competent bacteria [33–35]; in freshwater natural communities grown ex situ, respiration rates and organic substrate assimilation can vary in response to periodic infrared light exposure, suggesting that these variations are driven by AAnP [36]. Laboratory based studies of AAnP models show clear differences in global transcription patterns between cells cultured in the dark and in the light, some of these differences being directly connected to light-stress responses [37, 38]. Finally, studies on pure cultures of *Roseobacter* sp. OCH 114 and *Dinoroseobacter shibae* DFL12 under controlled conditions showed that these bacteria benefit from exposure to continuous light or dark-light cycles for their survival, although this survival benefit was never genetically linked to phototrophy [17, 39]. Despite these convergent indications, consistent evidence on a single experimental model, ranging from genetics to fitness measurements, is actually missing. Moreover, it is generally unclear how AAnP affects bacterial fitness under diel cycles and how facultative aerobic anoxygenic phototrophs navigate the tradeoff between costs and benefits of phototrophy.

Here, we investigate how a new freshwater model of AAnP-competent bacteria, *Porphyrobacter* sp. ULC335, responds to and benefits from diel dark-light cycles. When grown to stationary phase under dark-light alternance, *Porphyrobacter* sp. ULC335 presents rhythmic patterns of density variation that were not reported in most other species capable of AAnP. We explain these patterns by connecting them to variations in mortality and reproduction rates due to phototrophy, study their transcriptional correlates, and reveal their impact on fitness. In so doing, we contribute to a better understanding of the rhythmic patterns governing the physiology and lifestyle of bacteria prevalent in photic aquatic layers.

## Methods

Detailed methods are available in a “Supplementary Methods” file.

### Standard growth conditions

*Porphyrobacter* sp. ULC335 was grown in BG11 media [40] supplemented with 0.5 g/L tryptone, 0.25 g/L yeast extract, and vitamins. Standard batch growth took place at 30 °C with agitation (180 rpm). Growth in the Photon Systems Instruments MC1000 took place at 28 °C. Batch cultures for competition and survival experiments were done in 24-well plates at 28 °C with agitation.

### Pigment characterization

Cell pellets were extracted with 7:2 acetone-methanol for 5 min; the absorbance of the supernatant was measured in quartz cuvettes (Genesys 10 s, Thermo Scientific) or in 96-well plates (SpectraMax i3x, Molecular Devices). For Thin Layer Chromatography, 100 µl of extract were spotted onto a Silica 60 F_254_ plates (Merk, art. 5554). The plate was migrated in 8:0.75:0.2:0.8:0.25 petroleum ether:hexane:isopropanol:acetone:methanol.

### Transposon library

The plasmid pRL27 [41] was conjugated into *Porphyrobacter* sp. ULC335 and several mutants whose color differed from the wild type were isolated. The insertion sites were identified using the method previously published [41]. Four mutants are central to our study: mutant B^+^C^−^ (insertion in *crtI*, coding for the phytoene synthase, required for carotenoid production), mutant B^−^C^+^ (insertion in *bchH*, coding for a subunit of the magnesium chelatase, required for Bchl*a* synthesis), mutant B^−^C^−^ (insertion in *ispA*, coding for the farnesyl diphosphate synthase, required to synthesize a precursor of both Bchl*a* and carotenoids), mutant B^++^C^++^ (insertion in the *ppaA* gene coding for an AAnP regulator).

### Genome sequencing and annotation

The genomic DNA was purified using the Qiagen Genomic-tip 20/g kit and sequenced on PacBio Sequel instruments. The genome was assembled using PacBio *CCS* 4.1.0 for the read correction step and *Flye* 2.6 [42] for the assembly. The functional classification of the genome content was done using the online *blastKOALA* service with the genus *Porphyrobacter* as a reference.

### RNA-sequencing

RNA was extracted from 1 ml culture using TRIzol (Invitrogen) followed by purification on RNeasy mini-kit columns (Qiagen). RNA-sequencing was carried out at Novogene on Illumina NovaSeq systems after ribosomal depletion and in-house library construction. Reads were trimmed and filtered using *fastP* [43], mapped using *bowtie2* [44], and counted using *featureCounts* [45]. Comparison between wildtype and B^−^C^+^ strains was done using the *DESeq2* package [46]. Temporal analysis of individual genes was done using the *ImpulseDE2* package [47]. Each temporally regulated gene was assigned to a cluster using *hclust* (Euclidian algorithm, 15 clusters) on the Z-scores of the *rlog*-transformed count data for all four timepoints. The *ggVennDiagram* [48] and *ggplot* [49] libraries were used to plot the summary figures.

### Phylogenetic analysis

Fourteen protein sequences characteristic of the AAnP system from complete alphaproteobacterial genomes were concatenated and aligned using *muscle* 3.8.31 [50]; a phylogenetic tree was constructed using *iqtree* 2.1.1 [51] with default parameters (evolution model chosen automatically). In parallel, a set of conserved ribosomal proteins, elongation factors, and gyrases were retrieved and a phylogenetic tree was constructed following the same procedure. Strains were considered to encode the anoxygenic phototrophy gene cluster if they gave positive *blastp* matches with at least 13 out of the 14 proteins.

### Flow cytometry and fluorescence microscopy

Cells stained with LIVE/DEAD BacLight (Invitrogen) were analyzed by flow cytometry (BD Accuri C6 Plus). The zones corresponding to the dead, compromised, and live subpopulations were determined empirically based on the density distribution of live and 2.5% glutaraldehyde-fixed cells. Visualization and quantification were done with *R* [52] using *flowCore* [53]. The same cells were analyzed using fluorescence microscopy, where only dead and live subpopulations could be distinguished.

Cells stained with DyeCycle Orange (Invitrogen) were analyzed by flow cytometry. The coordinates of the highest density in the regions of the 1 N and 2 N peaks (green fluorescence) were determined empirically. The lowest density point between the 1 N and the 2 N peaks on the empirical density plot was used as a proxy for the “actively replicating” population.

### Survival and competition experiments

For survival experiments, late exponential phase cultures of wild type, B^−^C^+^, and B^++^C^++^ were diluted and grown under continuous light, continuous dark, or 12 h/12 h dark-light cycles in triplicates. At the beginning of stationary phase (48 h) and after four additional days (144 h), CFUs were determined and the survival rate (144 h vs 48 h) was calculated.

For competition experiments, exponential phase cultures of the two mutant strains were mixed with the wild type strain in equal proportions and grown under continuous light, continuous dark, or 12 h/12 h dark-light cycles in triplicates. The dual cultures were plated at 48 h and 144 h like for the survival experiment, and the ratios of the mutant to the wild type were calculated for both timepoints based on CFUs.

In survival and competition experiments, irradiance during light periods was 80 µmol/s/m^2^.

## Results

### Strain ULC335 is a representative of the *Porphyrobacter* genus, in which AAnP is ancestral

**Fig. 1:**
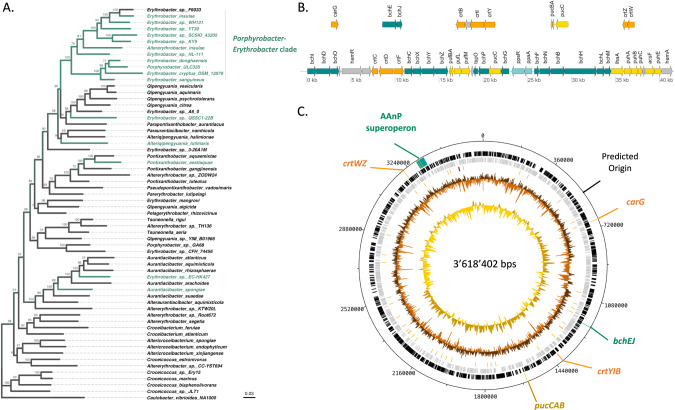
*Porphyrobacter* sp. ULC335 is an aerobic anoxygenic phototroph. **A** Simplified phylogenetic tree made from an alignment of concatenated core-genome proteins of Erythrobacteraceae rooted on *Caulobacter crescentus* (Caulobacterales); a tree comprising more strains can be found in Suplementary Fig. 4. *Porphyrobacter* sp. ULC335 branches within the *Porphyrobacter-Erythrobacter* clade, in which the anoxygenic phototrophy gene cluster is quasi-ubiquitous (branches in green). Scale bar: average number of substitutions per site. Numbers at the nodes are support values (percent of generated trees supporting the branch). **B** The aerobic anoxygenic phototrophy (AAnP)-associated genes found in *Porphyrobacter* sp. ULC335. The AAnP-associated genes are scattered in six loci, one major locus more than 40 kb in length, and five minor loci, comprising two or three genes. The color codes indicate the predicted functional categories of the products: dark green for proteins involved in bacteriochlorophyll *a* synthesis, yellow for structural proteins of the reaction centers, orange for proteins involved in carotenoid synthesis, light green for regulatory genes, gray for genes associated with the AAnP genes but whose function is not clear. **C** Schematic overview of the genome of *Porphyrobacter* sp. ULC335. The concentric circles represent, from inside out, the GC skew (yellow and gold), the GC content (light and dark brown), RNAs (red), genes encoded on the reverse and forward strand (gray and black), AAnP-related loci (same color code as in **B**), genome coordinates using the coding sequence of *dnaA* as a reference.

**Fig. 2:**
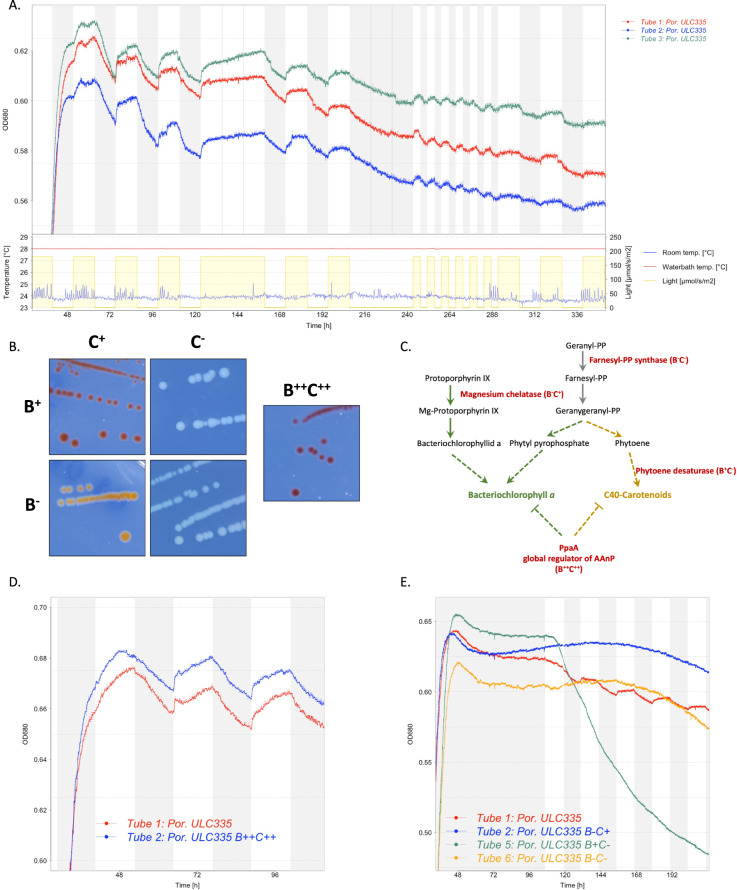
*Porphyrobacter* sp. ULC335 presents rhythmic growth patterns in stationary phase that are dependent on functional anoxygenic phototrophy. **A** Rhythmic variations in optical density (measured at 680 nm) of three replicate cultures of *Porphyrobacter* sp. ULC335 under dark-light alternance (light at 180 µmol/s/cm^2^). Light intensity, culture temperature (water bath), and room temperature are shown as evidence that density changes are from biological and not technical origin. Cultures were maintained in 80 ml tubes in a device adapted for the culture of cyanobacteria or algae. The rhythms do not present persistence under continuous light (120 h–156 h) or continuous darkness (204 h–240 h), and follow light variation seamlessly when the period is shorter than 24 h (240 h–288 h). **B** Colony phenotype of *Porphyrobacter* sp. ULC335 (B^+^C^+^) and four transposon mutants grown on BG11-P-agar in the dark. The color of colonies are in agreement with the absorbance profiles of liquid cultures (Suplementary Fig. 12). **C** Simplified pathway of carotenoid and bacteriochlorophyll *a* biosynthesis. In red, the genes in which the transposon is inserted for each of the mutant strains. Ticked lines represent multiple synthesis steps (enzymatic reactions) or multiple convergent effects (regulators). **D** Rhythmic variations in optical density (measured at 680 nm) of the wild type compared with the bacteriochlorophyll *a* and carotenoid overproducer strain. No significant difference in pattern is apparent between the two strains. The culture conditions are similar to (**A**). **E** Variations in optical density (OD, measured at 680 nm) of the wild type compared with the three bacteriochlorophyll *a* and/or carotenoid mutant strains. B^−^C^+^ and B^−^C^−^ did not present any rhythmic growth pattern; B^+^C^−^ presented slight variations in slope between dark and light phases, the speed of the OD decline increasing during light phases. Because B^+^C^−^ was strongly inhibited by light, the cultures were maintained under continuous darkness until stationary phase, when a 12 h/12 h dark-light alternance illumination regime was started.

In the course of a study on a bacterial community associated with a single cyanobacterial species (ULC335) [54] (Suplementary File 1), we isolated a nonmotile dark-red colony-forming bacterial strain which, based on its 16S ribosomal RNA sequence, was assigned to the *Porphyrobacter-Erythrobacter* group (Erythrobacteraceae genus I–VI) [55]. The strain encoded an allele of the *pufM* gene, coding for a light-harvesting complex subunit, and readily produced, under standard aerobic culture conditions, bacteriochlorophyll *a* (hereafter “Bchl*a*”) (Suplementary Figs. 1 and 2), suggesting that the strain was capable of AAnP [56, 57].

**Fig. 3:**
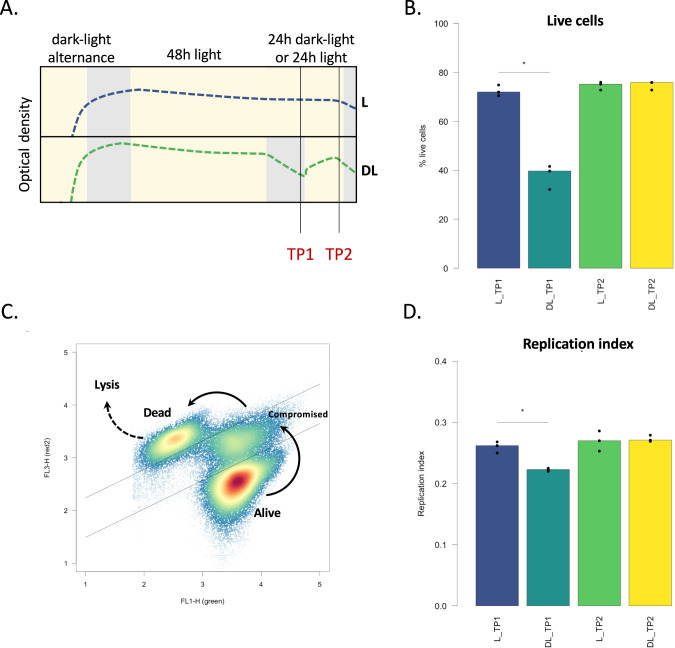
Variations in optical density correlate with differences in cell death and replication rates. **A** Experimental design. Six *Porphyrobacter* sp. ULC335 cultures were maintained in parallel under dark-light alternance up to stationary phase to allow for carotenoid and bacteriochlorophyll *a* accumulation; then, the cultures were exposed for 48 h to continuous light, before three of the six cultures were switched to the dark for 12 h and back again to light for another 12 h. Two series of samples were taken, one at the end of the dark period and one 8 h after return to light, and analyzed using fluorescence microscopy and flow cytometry. **B** Living cells as determined using fluorescence microscopy on Live/Dead stained cells. The proportion of living cells dropped after the dark phase and returned to baseline after exposure to light. At least 500 cells were counted per replicate. **C** Dead, compromised, and alive subpopulations as observed using flow cytometry after Live/Dead staining. The arrows indicate the probable path from alive to compromised to dead cells, as membrane permeability increases; lysed cells have released their cellular content, including DNA, and cannot be observed using flow cytometry. **D** The proportion of replicating cells, as determined by flow cytometry using DyeCycle Orange stain, decreased during the dark phase and returned to baseline after reexposure to light. A total of 40'000 cells was analyzed. The calculation of the replication index is explained in the methods; see Suplementary Fig. 19 for a more detailed representation of flow cytometry data. Amount of light during light periods: 150 µmol/s/m^2^. Asterisks indicate statistically significant differences (bilateral Student’s *t*-test, *p*-value < 0.05).

Bacteria belonging to the *Porphyrobacter-Erythrobacter* group are ubiquitous in aquatic environments and often found associated with Cyanobacteria [58–61]. While many genera among alphaproteobacteria contain members capable of anoxygenic phototrophy (Suplementary Fig. 3), the *Porphyrobacter-Erythrobacter* group is one of the few taxonomic units in which the AAnP gene cluster is most likely ancestral and present in the vast majority of sequenced strains (Figs. 1A, SFig. 4). Moreover, very few members of the *Porphyrobacter-Erythrobacter* have lost the gene cluster, which indicates that it consistently provides a benefit. Noteworthy, the dominant mode of transmission of the genes coding for anoxygenic phototrophy within alphaproteobacteria, including within the *Porphyrobacter-Erythrobacter* group, is vertical, suggesting that the gene cluster has extensively co-evolved and co-adapted with the core genome (Suplementary Figs. 5 and  6). On this basis, we consider the *Porphyrobacter-Erythrobacter* group as an excellent model to study the effects of long-term adaptation to AAnP.

To further characterize *Porphyrobacter* sp. ULC335, we sequenced its genome, obtaining a single closed circular sequence of 3.6 Mbps. Besides the typical genetic complement of free-living alphaproteobacteria (Suplementary Fig. 7A, B), it encoded a complete AAnP gene cluster comparable to the one found in sequenced members of the *Porphyrobacter-Erythrobacter* group (Fig. 1B) [15] and no less than five blue-light responsive proteins including a BLUF domain [26, 62] (Suplementary Fig. 7C). Some of the genes coding for the carotenoid synthesis pathway are found in the AAnP gene cluster (*crtC, crtD, crtF*), others in secondary loci (*crtZ, crtW; crtB, crtI, crtY; crtG)* (Fig. 1B, C). Thin layer chromatography revealed that, besides Bchl*a* and its intermediates, *Porphyrobacter* sp. ULC335 synthesizes a number of red and yellow pigments (Suplementary Fig. 8). *Porphyrobacter* sp. ULC335 does not encode a full carbon fixation pathway and is unlikely to grow autotrophically. However, like *Erythrobacter* sp. NAP1, it produces key enzymes of the 3-hydroxypropionate bicycle pathway for carbon fixation, which would give it the capacity to co-assimilate CO_2_ while using glycolate, glyoxylate, or acetate, among others, as carbon sources (Suplementary Fig. 9) [19, 63]. Altogether, these data suggest that *Porphyrobacter* sp. ULC335 could make use of light to spare organic substrates under nutrient limitation [20].

### In stationary phase, *Porphyrobacter* sp. ULC335 presents light-induced rhythmic growth patterns dependent on a functional photosystem

When monitored under 12 h/12 h dark-light cycles, *Porphyrobacter* sp. ULC335 presented, during stationary phase, strong variations in optical density in response to light in a range of light intensities (Fig. 2A, Suplementary Fig. 10). The bacterial density increased during the light phase and decreased during the dark phase; the rhythms strictly followed the photoperiod and did not persist under constant light or darkness (Fig. 2A, Suplementary Fig. 11), which suggests that they were not driven by a circadian clock but by external light variations. We hypothesized that such variations in optical density could be a consequence of the phototrophic activity of the organism, although, to our knowledge, such conspicuous rhythms have not been reported so far in bacteria capable of AAnP in the absence of additional phototrophic systems. To test this hypothesis, we generated a Tn5 transposon mutant library of the strain and screened for color variations which would betray an alteration of Bchl*a* or carotenoid content. We obtained four mutants of interest (Fig. 2B): strain B^+^C^−^ produced Bchl*a* but no carotenoids (insertion in *crtI*, coding for the phytoene synthase), strain B^−^C^+^ produced carotenoids, although less than the wild type, but no Bchl*a* (insertion in *bchH*, coding for a subunit of the magnesium chelatase required for Bchl*a* synthesis), strain B^−^C^−^ produced neither Bchl*a* nor carotenoids (insertion in *ispA*, coding for the farnesyl diphosphate synthase, required to synthesize a precursor of both Bchl*a* and carotenoids), strain B^++^C^++^ overproduced both Bchl*a* and carotenoids, especially in rich medium and under continuous light (insertion in the *ppaA* gene coding for an AAnP regulator) (Fig. 2C; Suplementary Fig. 12). Of the four mutants, only the B^++^C^++^ strain presented rhythmic growth similar to the wild type strain (Fig. 2D). By contrast, strains B^−^C^−^ and B^−^C^+^ did not present any periodic optical density variation (Fig. 2E). The strain B^+^C^−^ was very sensitive to light: as soon as light was switched on the density started decreasing and continued during the subsequent cycles (Fig. 2E); upon light exposure, the viability of cells, as quantified by Life/Dead staining and CFUs, decreased by at least two orders of magnitude (Suplementary Fig. 13). This suggests that, in the absence of carotenoids, Bchl*a* production might be toxic to the cells, especially in the presence of light. Overall, these data indicate that the rhythmic density variations in response to light depend on a functional reaction center including both Bchl*a* and carotenoids.

### The optical density variations in response to light correlate with changes in cell viability and DNA replication

To further investigate the origin of these rhythms, we sought to determine what happens to *Porphyrobacter* sp. ULC335 cells when they are exposed to darkness. To do so, we sampled six parallel cultures of *Porphyrobacter* sp. ULC335 grown to stationary phase and maintained under continuous light for two days; three of the cultures were switched to the dark for 12 h before sampling (Time Point 1 [TP1]) and switched back to light immediately after sampling (dark treatment); a second sample of all six tubes was taken eight hours after the switch back to light (TP2) (continuous light treatment) (Fig. 3A, Suplementary Fig. 14). Using spectrophotometry and flow cytometry, we did not find any difference in pigment content, cell morphology or aggregation between the treatments (Suplementary Fig. 15). However, using Live/Dead staining, we observed a clear difference in the proportion of living and compromised (permeable) cells between the two treatments: while 70% cells cultured under continuous light were viable and non-compromised, based on fluorescence microscopy, this proportion dropped to 40% after 12 h in the darkness; interestingly, it was enough for the latter cultures to be exposed back to light for 8 h to restore the proportion of viable cells to original levels (Fig. 3B). Colony forming units confirmed this trend (Suplementary Fig. 16). Flow cytometry revealed that three subpopulations coexist in the cultures, independently of the treatment: viable noncompromised cells, compromised (permeable) cells, and dead cells (Fig. 3C; controls: Suplementary Fig. 17). Like the “dead” subpopulation, the “compromised” subpopulation was significantly larger in the cultures exposed to 12 h of darkness than in the cultures exposed to continuous light (Suplementary Fig. 18). Moreover, by analyzing the DNA content of the cells, we found that the frequency of cells undergoing DNA replication was significantly lower in the cultures exposed to darkness than in the cultures kept under continuous light; upon reexposure to light, the two populations were indistinguishable again (Fig. 3D; Suplementary Fig. 19). Taken together, these results suggest that the life history of *Porphyrobacter* sp. ULC335 follows alternate phases in response to illumination conditions: during dark-phases, cells slow down replication and a significant proportion of them dies; during light-phases, part of the surviving cells replicate and divide, and this, possibly along with the partial recovery of some compromised cells, leads to the restoration of previous viability levels in the population.

### In *Porphyrobacter* sp. ULC335, direct responses to light and responses to electron flow underlie rhythmic physiology

To understand the physiological correlates of this behavior, we sequenced the full transcriptome of stationary phase cultures under 12 h/12 h darklight cycles. We were particularly interested in transcriptional responses upon light phase transition, so we sampled one hour before (Late Night, “LN”) and after (Early Day, “ED”) the switch to light, one hour before (Late Day, “LD”) and after (Early Night, ”EN”) the switch to darkness. In addition, we treated in parallel the wild type strain and the arrhythmic phototrophy mutant B^−^C^+^ in order to be able to discriminate between direct responses to light (independent of Bchl*a*) and responses due to the electron flow generated by phototroph.

**Fig. 4:**
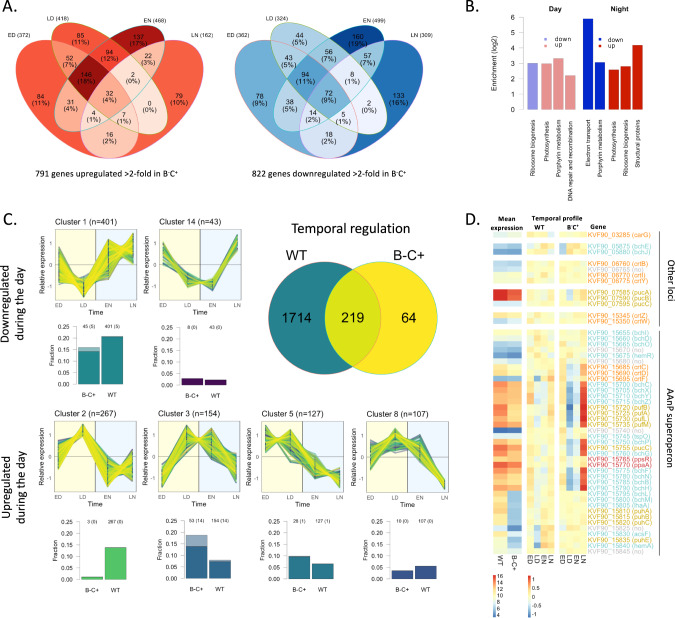
Diel dark-light cycles drive extensive transcriptional rhythms in *Porphyrobacter* sp. ULC335. **A** Summary of the experiment: number of genes upregulated (right) or downregulated (left) in the Bchl*a* null mutant compared to the wild type at different timepoints (ED: early day; LD: late day; EN: early night; LN: late night). **B** KEGG categories enriched among differentially regulated genes in the Bchl*a* null mutant compared to the wild type; ED and LD on one side and EN and LN on the other side are considered together in this analysis. **C** The figure summarizes information about the temporal regulation of genes in the wild type strain and its Bchl*a* null mutant. The Venn diagram indicates the number of temporally regulated genes in the two strains. The major clusters of genes showing a consistent light-response pattern in either of the two strains are presented based on their temporal profile (upper plot; the datapoints represent the relative transcription rates [the Z-scores of the rlog-transformed count data]) and the fraction they represent among temporally regulated genes in both strains (lower plot; light colors represent genes falling in the same cluster in the wild type and the Bchl*a* null mutant). The same data split by strain is presented in Suplementary Fig. 20. **D** Heatmap of gene expression for the AAnP-associated genes. The first two columns show the relative expression of each gene in the two strains; the next eight columns represent the variation over time of the two strains. The gene number and name are shown in cyan for Bchl*a* biosynthesis genes, gold for reaction center genes, orange for carotenoid biosynthesis genes, and red for regulators; gray is used for genes whose function is dubious.

Disrupting photosynthesis had massive consequences at the transcriptional level. Among protein-coding genes, 1533 out of 3418 were differentially regulated more than two-fold at least at one of the four timepoints in the phototrophy mutant, about half being downregulated and half being upregulated (Fig. 4A; Suplementary File 2). The time of day had a strong influence on the divergence of the transcriptional profile between the two strains. The number of differentially regulated genes between the two strains differed the most at the EN time point and differed the least at the LN time point, suggesting that, in the prolonged absence of light exposure, the physiologies of the two strains tended to converge. Globally, the mutant and the wild type seem to time transcription differently. Genes more transcribed in the mutant compared to the wild type were most abundant during the day (and EN), while genes less transcribed in the mutant compared to the wild type were most abundant during the night. Among genes downregulated in the mutant, functions linked to photosynthesis, including porphyrin metabolism, and ribosome biogenesis were enriched, especially during the day; genes upregulated in the mutant included categories linked to DNA repair, translation, and energy metabolism (Fig. 4B; Suplementary File 3). This indicates that the transcriptional investment into energy metabolism, especially photosynthesis, and translation machinery is modified, both in terms of timing and intensity, in the phototrophy mutant compared to the wild type.

We then identified genes differentially regulated over time (Impulse DE algorithm, *p*-value < 0.01) in either the wild type or the B^−^C^+^ strain, clustered them by temporal profile and compared the different sets of genes between the two strains (Suplementary File 4; Suplementary Fig. 20). Again, photosynthesis disruption had a huge impact: only 283 genes (8% of the genome) varied over time in the B^−^C^+^ strain, compared to 1933 genes (56% of the genome) in the wild type strain. This indicates that functional AAnP not only provides energy to the cells, but also drives a pervasive rhythmic physiology that is entirely absent in the phototrophy mutant. More than half of the genes temporally regulated in the wild type strain show opposite expression trends during daytime and nighttime. Four gene clusters (655 genes in total) are consistently upregulated during the day and downregulated during the night (Fig. 4C, lower part), while two clusters (444 genes in total) are downregulated during the day and upregulated during the night (Fig. 4C, upper part). Overall, this suggests that the transcriptional profile, and consequently the physiology, of *Porphyrobacter* sp. ULCC335 presents a marked contrast between day and night involving opposed regulation of more than 1000 genes (31% of the genes). Genes downregulated during the day and upregulated during the night are enriched for the AAnP pathway, ribosome biosynthesis, and oxidative phosphorylation, including the electron transfer chain; genes upregulated during the day and downregulated during the night include transporters, energy metabolism and especially genes involved in the 3-hydroxypropionate bicycle and possibly implicated in CO_2_ co-assimilation (Suplementary File 5). This suggests that the night is dedicated to replenish Bchl*a* and the ribosomal content of the cells, while nutrient assimilation might be privileged during the daytime.

Like in other AAnP-competent bacteria, genes involved in Bchl*a* biosynthesis and components of the light-harvesting complexes tended to be downregulated during the day and upregulated at night in both the wild type and the Bchl*a* null mutant strain (Fig. 4D; Suplementary Fig. 21). However, the amplitude of expression changes across the four timepoints was attenuated in the wild type strain compared to the Bchl*a* null mutant. In B^−^C^+^, more than half of the expressed genes presented a four-fold difference in expression between late night (maximal expression) and late day (minimal expression), while a variety of attenuated and shifted profiles was represented in the wild type. These differences in the expression of AAnP-related genes suggest that genes which mainly respond to light cues in the B^−^C^+^ strain are tuned, in the wild type, via mechanisms sensitive to energy flow which are only active when AAnP is functional.

Overall, our transcriptome analysis revealed that diel light cycles have a major impact on the transcriptional program and physiology. The activity of the cells differs markedly between the daytime and the nighttime, and there is a clear physiological shift between early and late timepoints in either day or night. Consistent with our observations on optical density, inactivation of AAnP hampers rhythmic transcription and leads to a transcriptional profile that is most similar to the one presented by the AAnP-competent strain at the end of the night, when the benefits of phototrophy wear off.

### Phototrophy provides a survival benefit in stationary phase that correlates with Bchl*a* production

*Porphyrobacter* sp. ULC335 cultures present oscillations in density in stationary phase that correlate with changes in mortality and reproduction rates. However, it is unclear how these variations affect the overall survival of the bacteria, especially in view of the absence of any density variation in Bchl*a* null strains (B^−^C^+^ and B^−^C^−^). We took advantage of the set of mutants we had generated (Fig. 2B, C) to ask whether AAnP actually provides some fitness advantage to *Porphyrobacter* sp. ULC335 depending on the illumination regime (DL: 12 h/12 h Dark-Light alternance, D: constant Dark, L: constant Light).

**Fig. 5:**
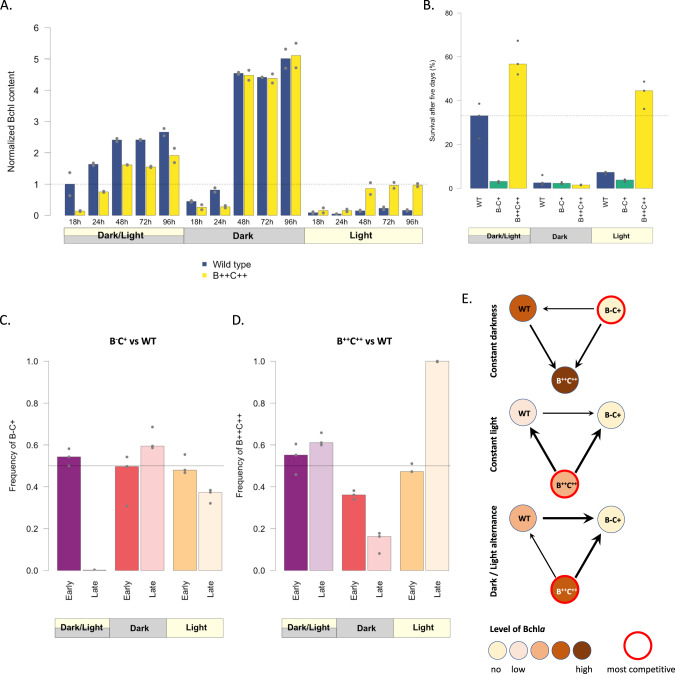
Phototrophy provides a major benefit to *Porphyrobacter* sp. ULC335 in stationary phase. **A** Bchl*a* content at different timepoints during batch culture. The barplots represent the absorbance at 770 nm (Bchl*a*) corrected by the optical density of the culture (average of two replicates), normalized so that the average of the first timepoint of the wild type strain equals 1. The wild type strain is shown in blue, the Bchl*a* overproducer (B^++^C^++^) in yellow. The experiment was done under 12 h/12 h dark-light cycles, under continuous darkness, or under continuous light. **B** Percent survival, based on colony forming units, of the wild type, the Bchl*a* null mutant (B^−^C^+^) and the Bchl*a* overproducer (B^++^C^++^) after five days in stationary phase. The experiment was done under 12 h/12 h dark-light cycles, under continuous darkness, or under continuous light. The baseline is taken in early stationary phase (48 h of growth). **C**, **D** Outcome of competitions between the wild type and each of the two mutants after five days in stationary phase. The two competing strains were mixed in equal proportions after they had reached stationary phase (48 h of growth). **E** Figure summarizing the competitiveness of each strain under different illumination conditions. The inner color of the circles varies with relative Bchl*a*/carotenoid content; the most competitive strains are outlined in red; the relative competitiveness is represented by arrows of three different thicknesses.

While AAnP did not provide a significant advantage in exponential up to early stationary phase (Suplementary Figs. 22 and 23), survival after 96 h spent in stationary phase was strongly affected by the genotype and the illumination regime. This is consistent with the accumulation pattern of Bchl*a* and carotenoids, whose content increases a lot between 24 h and 48 h of growth, especially in the D and DL treatments, in the two strains capable of AAnP (Fig. 5A). When AAnP was impossible, either in the absence of light or in the absence of Bchl*a* (B^−^C^+^ mutant), the CFUs decreased by about two orders of magnitude leading to survival rates around or below 5% (Fig. 5B). The capacity to produce energy through AAnP helped the wild type and B^++^C^++^ strains survive better under the DL regime, reaching 35% and 60% survival respectively. Under continuous light, again, the wild type and B^++^C^++^ strains survived better than the Bchl*a* null mutant, but mortality was nonetheless much higher than under the DL regime, presumably because Bchl*a* accumulation was impaired (Fig. 5A). These results demonstrate that AAnP provides a survival advantage to *Porphyrobacter* sp. ULC335 that depends on both growth phase and illumination regime, and partially correlates with Bchl*a* accumulation. In our strain, AAnP is therefore not primarily used to supplement active heterotrophic growth, but to enhance survival during periods of starvation, especially under DL illumination.

To complement these conclusions, we ran competition experiments in which the wild type strain and a competitor strain (either B^−^C^+^ or B^++^C^++^) were grown together in batch under a DL, D, or L regime. We sampled the cultures at 48 h (start of stationary phase) and at 144 h (late stationary phase), and compared the ratios between the strains. The competitiveness of the strains varied depending on the light regime in predictable ways (Fig. 5C, D). In the absence of light (D), the Bchl*a* null mutant outcompeted both Bchl*a* producers, and the wild type strain outcompeted the B^++^C^++^ strain. In the presence of light, under both the DL and L regimes, the B^++^C^++^ strain outcompeted the wild type strain which, in turn, outcompeted the Bchl*a* null mutant. Altogether, these results indicate that, in *Porphyrobacter* sp. ULC335, AAnP has a significant cost, including in stationary phase, but that the fitness advantages it provides as soon as light is present—even when light is continuous and prevents the efficient accumulation of Bchl*a*—warrants its maintenance in natural populations.

It stands out that, in our experimental conditions, the wild type strain is less fit than the Bchl*a* and carotenoid overproducer B^++^C^++^ whenever light is present. This suggests that, in the natural environment of *Porphyrobacter* sp. ULC335, the photoperiod is on average lower than 12 h or that the regular diel dark-light alternance can often be interrupted by periods of continuous accidental darkness (Fig. 5E).

## Discussion

In this work, we have isolated and described a new *Porphyrobacter* strain, which we found associated with a freshwater cyanobacterium (*Snowella* sp.). In stationary phase, this strain presented rhythmic variations in optical density in response to dark-light cycles, that we correlated with phases of high mortality and low reproduction in the darkness and phases of lower mortality and higher reproduction in the light. This behavior is dependent on Bchl*a* production and a functional reaction center, including carotenoids contributing to photoprotection and light-harvesting. Underlying the cycles, we found pervasive rhythmic transcription patterns arising from direct responses to light, possibly driven by any of the five BLUF-domain containing proteins encoded in the genome, and indirect responses to increased electron flow and oxidative stress caused by phototrophic activity. Production of Bchl*a* was found to be costly under continuous darkness and inefficient under continuous light; it provides maximal survival benefits in stationary phase under dark-light cycles, indicating that the strain is adapted to diurnal light cycles. So overall, in this study, we described a distinct bacterial rhythmic behavior, explained it in terms of fluctuations in death and reproduction rates at the cellular level, partially elucidated the transcriptional patterns that underlie it, and linked it to a survival benefit, providing a rationale for its evolution.

Based on average nucleotide identity with known genomes, *Porphyrobacter* sp. ULC335 is likely a new species in the *Porphyrobacter-Erythrobacter* clade (Suplementary Fig. 24). While *Porphyrobacter* sp. ULC335 probably obtains most of its energy, reductive power, and carbon from organic substrates, it can use phototrophy to supplement heterotrophic growth and to ensure survival when organic substrates become limiting like other AAnP-competent bacteria [11]. Because of the phylogenetic prevalence of AAnP in the clade, these organisms are expected to have evolved ways to tune their physiology to nutrient and light fluctuations, as evidenced by the high numbers of blue-light sensing proteins encoded in the genome of *Porphyrobacter* sp. ULC335 together with homologs for PpsR, AppA, and FnrL, known to integrate phototrophy with metabolism [64, 65]. Because *Porphyrobacter* strains are frequently associated with filamentous or colonial Cyanobacteria [58, 66, 67], their physiology could also indirectly follow the light and circadian clock-driven life cycle of their host like it has been suggested for other Cyanobacteria-associated heterotrophs [68]. This makes members of the *Porphyrobacter* genus excellent candidates for the study of bacterial rhythmic behaviors and interactions.

The conspicuous light-driven oscillations in optical density we detected in batch cultures of *Porphyrobacter* sp. ULC335 have never been reported or studied so far in AAnP-competent bacteria, with the exception of *Sphingomonas glacialis* AAP5, recently isolated from an alpine lake, which combines retinalophototrophy and chlorophotoptrophy [69]. In our case, the oscillations involve an increase in density during light phases and a decrease in density during dark phases. Interestingly, the net difference in optical density per 24-h period is almost null (Suplementary Fig. 10) suggesting that the biomass lost during dark phases is regained during light phases. The full oscillatory pattern is strictly dependent on both Bchl*a* and carotenoids; however, when Bchl*a* is produced in the absence of carotenoids, light phases trigger a sharp decrease in optical density that hardly slows down during dark phases (Fig. 2E, Suplementary Fig 13). This suggests that Bchl*a*, or some derivative of it, might become toxic, for instance by generating reactive oxygen species, in the presence of light and cause cell lysis, including in a delayed way during the dark phase [22, 70, 71]. We showed that the decrease in optical density observed during dark phases correlates with an increase in the frequency of membrane-compromised and non-viable cells and a decrease in the frequency of replicating cells in the bacterial population. Based on the decrease in optical density, which corresponds to about 2 to 3% of the total biomass, it is likely that only a fraction of the 20% to 40% of compromised or dead cells (see Fig. 3B) actually lyse during the night. The decrease in compromised or dead cell proportion during the light phases is therefore most likely due to membrane repair and, to some extent, to continued lysis of affected cells, compensated by cell multiplication. Overall, these observations suggest that AAnP functions as a double edge sword: when exposed to light, AAnP photosystems sustain energy production and recycling of organic compounds; during dark phases, however, when energy becomes scarce, a reaction consecutive to Bchl*a* exposure to light leads to membrane permeabilization and cell lysis; during the following light phase, the population recovers both in terms of membrane integrity and in terms of viability. It is however unclear whether this recovery is mostly due to compromised cells being able to repair their membrane or to the renewed growth of the surviving population based on nutrients released by lysing cells.

Physiological rhythms in bacteria and especially in heterotrophs remain understudied. Our RNA-sequencing data set reveals that, when AAnP is active, the alternance of dark and light phases affects the transcription of more than 50% of the genes. The transcripts responding to light in the absence of Bchl*a* are only a minority of at most 10%. This indicates that physiological rhythms are pervasive in AAnP-competent bacteria and most likely generated through complex interactions between direct light responses (via light sensors), and indirect responses to light stress, redox and energy state, metabolism, cell-cycle, etc. About half of the genes are consistently upregulated or downregulated by light. This suggests that there is a degree of temporal separation of cellular activities between day and night in these bacteria. Night-activated genes include many genes connected to translation, especially components of the ribosome and translation complexes, as well as genes involved in phototrophy like in other AAnP-competent bacteria [37, 38]. This suggests that dark phases are dedicated to the replenishment of components essential for light-harvesting and cellular functioning; they tend to limit, however, active DNA replication as observed through flow cytometry. By contrast, light phases promote the transcription of genes involved in energy metabolism and protection against oxidative light stress, but also key genes in DNA replication (*dnaA* which falls in cluster 2, among genes maximally expressed during the day) and cell division (*ftsZ*, which falls in cluster 4, among proteins maximally expressed by the end of the day); these genes and functions would stimulate DNA replication and cell division, supporting our flow cytometry data (Fig. 3D).

Despite a few precursory studies, the evolutionary benefit of AAnP has not benefited from much attention. Previous research has found that exposure to light increases survival of *Roseobacter* and *Dinoroseobacter* strains in stationary phase, but without formal demonstration that this benefit can be traced back to AAnP [17, 39]. Here we provide, based on manipulation of genotypes and growth environments, an in-depth assessment of costs and benefits of AAnP in terms of its effect on survival and fitness in stationary phase. We find that Bchl*a* production has a significant cost under constant darkness, where Bchl*a* producers get outcompeted by nonproducers. By contrast, under continuous light or alternance, Bchl*a* producers outcompete nonproducers. Under continuous light and, to a lesser extent, under light alternance, strains producing higher levels of Bchl*a* and/or carotenoids get a strong selective advantage. This suggests that the regulation of AAnP in the wild type *Porphyrobacter* sp. ULC335 is adapted to dark-light cycles with a photoperiod lower than 12 h or to environments where cells can be accidentally cut off from light by opaque bodies or move out of the photic zone.

### Supplementary information

Supplementary Figures and methods

Supplementary File 1

Supplementary File 2

Supplementary File 3

Supplementary File 4

Supplementary File 5

## Data Availability

The *Porphyrobacter* sp. ULC335 genome is publicly available on the NCBI repository (SAMN19986570, GCF_025917005.1). The RNA-sequencing data is publicly available on the NCBI Gene Expression Omnibus (GEO) repository (GSE245047).
