# Supplementary material for: Diurnal cycles drive rhythmic physiology and promote survival in facultative phototrophic bacteria
Source: ISME Commun. 2023 Nov 24;3:125. doi: 10.1038/s43705-023-00334-5 (PMC10674011; doi:10.1038/s43705-023-00334-5)
Supplement: Supplementary file 1 — Supplementary Figures and methods [file 43705_2023_334_MOESM1_ESM.pdf]

Diurnal cycles drive rhythmic physiology and promote survival in facultative phototrophic bacteria

Camille Tinguely, Mélanie Paulméry, Céline Terrettaz, Diego Gonzalez

Supplementary Figures

and

Supplementary Methods

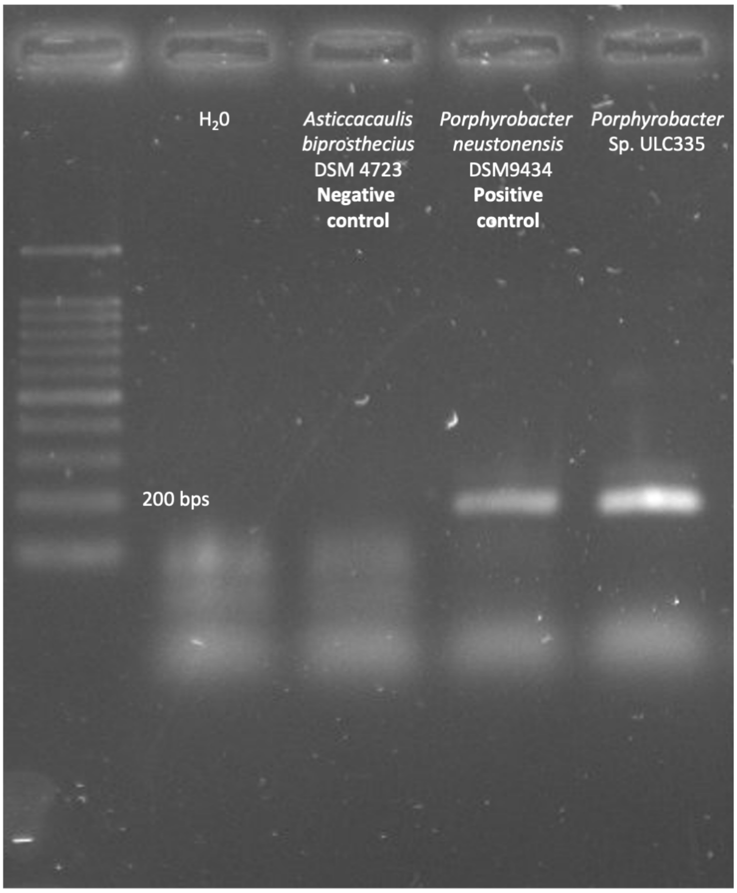

SFigure 1: The genome of *Porphyrobacter* sp. ULC335 encodes the *pufM* gene. The figure shows the results of a PCR with primers pufM-F and pufM-R detecting the *pufM* gene (coding for a structural component of the reaction center). Like the type species *Porphyrobacter neustonensis, Porphyrobacter* sp. ULC335 gives a positive signal.

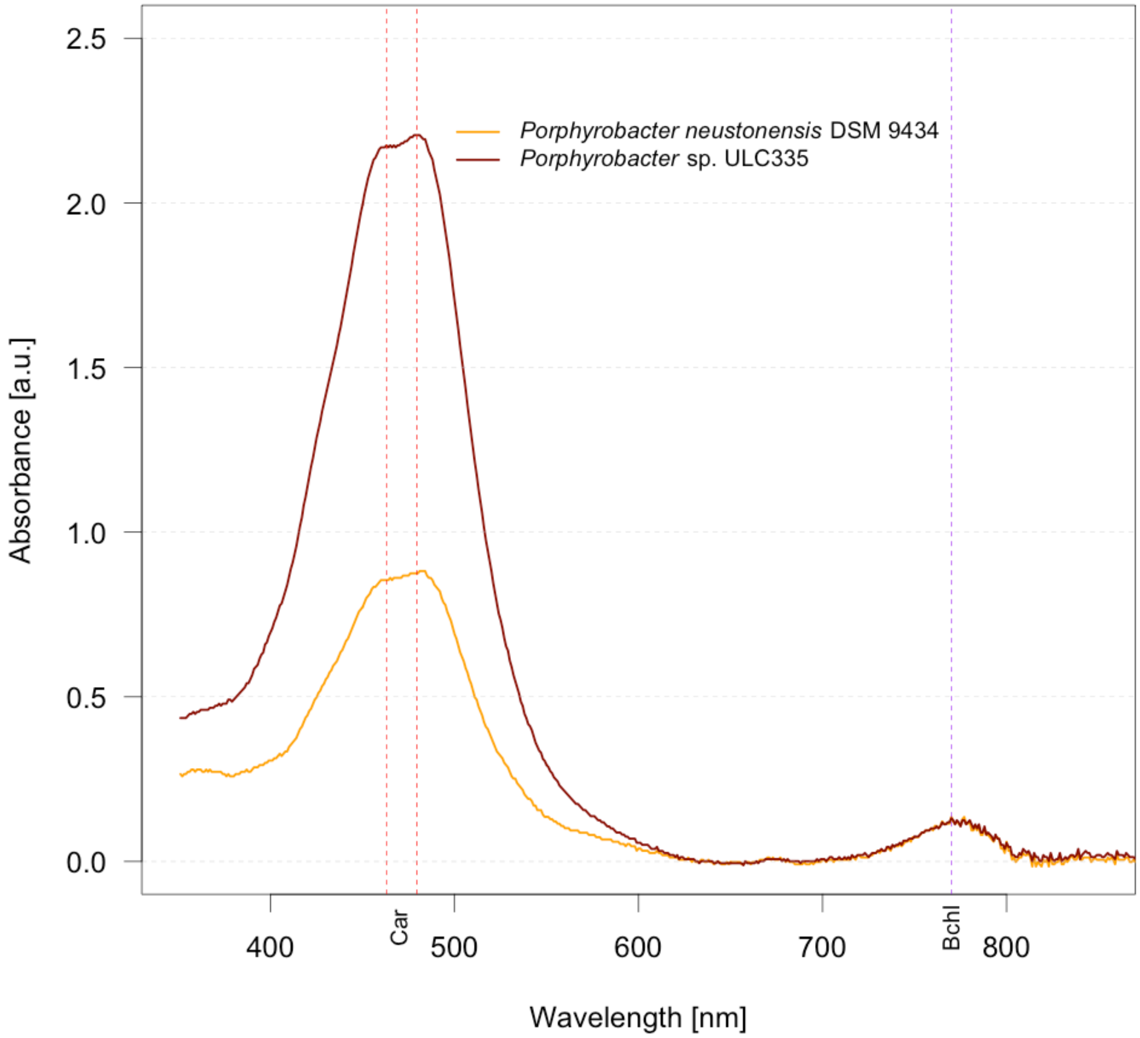

SFigure 2: *Porphyrobacter* sp. ULC335 produces pigments whose absorbance is consistent with carotenoids and bacteriochlorophyll *a*. The figure shows the absorbance profile of 7:2 acetone-methanol extracts of 48-hours cultures of *Porphyrobacter neustonensis* and *Porphyrobacter* sp. ULC335. In both species, peaks are found around 460 and 480 nm (carotenoids), and around 770 nm

(bacteriochlorophyll *a*). *Porphyrobacter* sp. ULC335 is noticeably more pigmented than *P. neustonensis.* Car: maxima for carotenoids; Bchl: maximum for bacteriochlorophyll *a.*

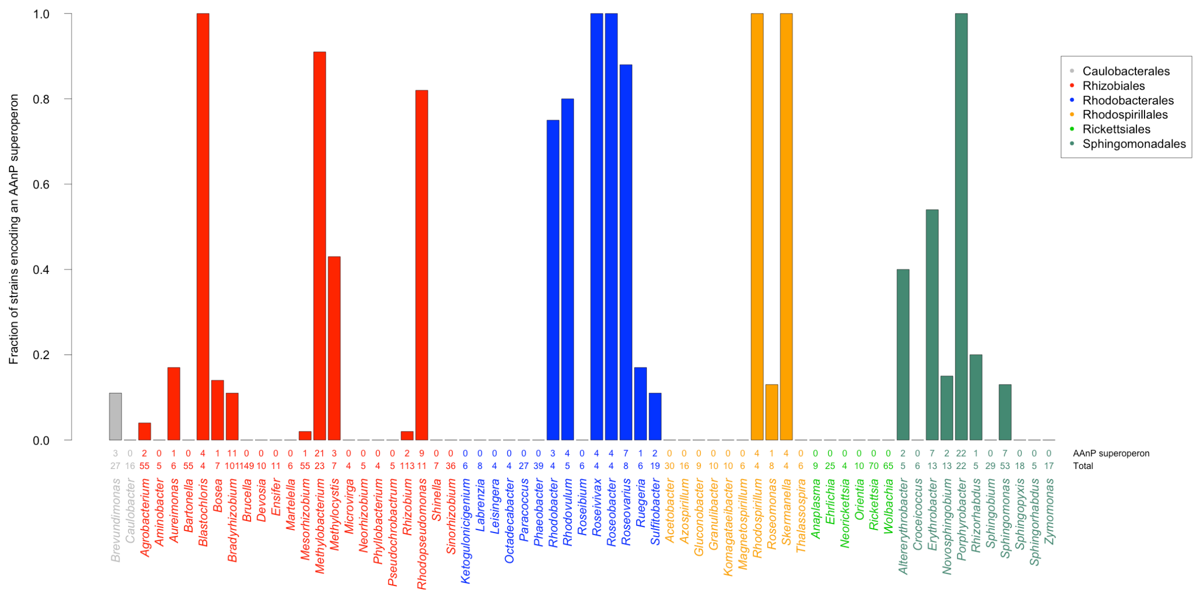

SFigure 3: Fraction of genomes per genus within alphaproteobacteria encoding the anoxygenic phototrophy superoperon. Genomes encoding at least 13 out of 14 signature proteins of the anoxygenic phototrophy superoperon were considered positive. Only genera including at least four full genomes were considered.

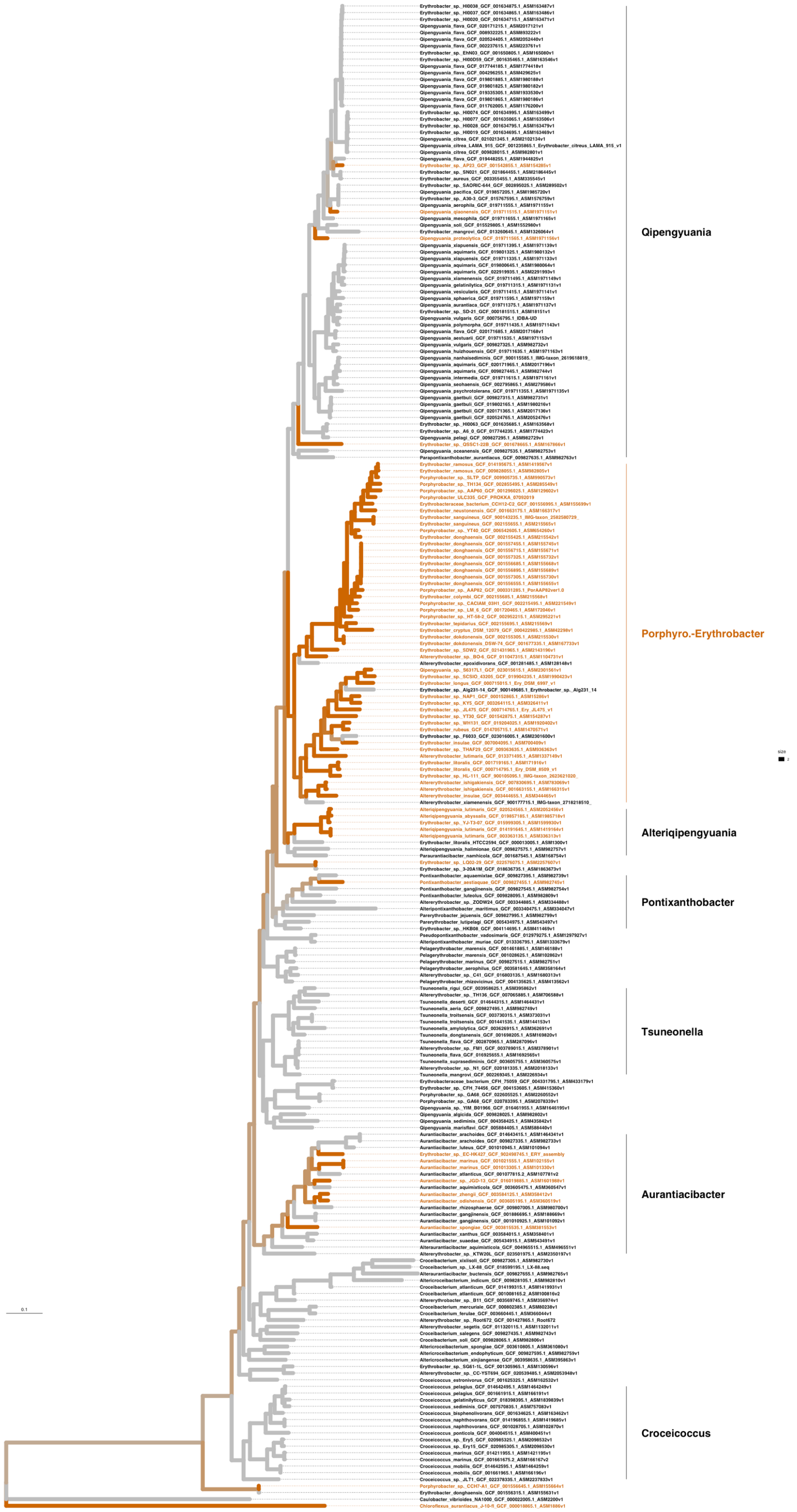
Qipengyuania
Porphyro.-Erythrobacter
Alteriqipengyuania
Pontixanthobacter
Tsuneonella
Aurantiacibacter
Croceicoccus
0.1

SFigure 4: Presence of the anoxygenic phototrophy superoperon within Erythrobacteraceae. The phylogenetic tree was constructed from a set of conserved core proteins from all genomes of Erythrobacterales listed on the NCBI repository. The anoxygenic phototrophy operon was considered present in the genome of at least 13 out of 14 signature proteins were found in it through a *blastp* search. The superoperon is clearly overrepresented in the *Porphyrobacter-Erythrobacter* clade. Scale bar: average number of substitutions per site. Bacteria encoding the anoxygenic phototrophy operon are highlighted in orange.

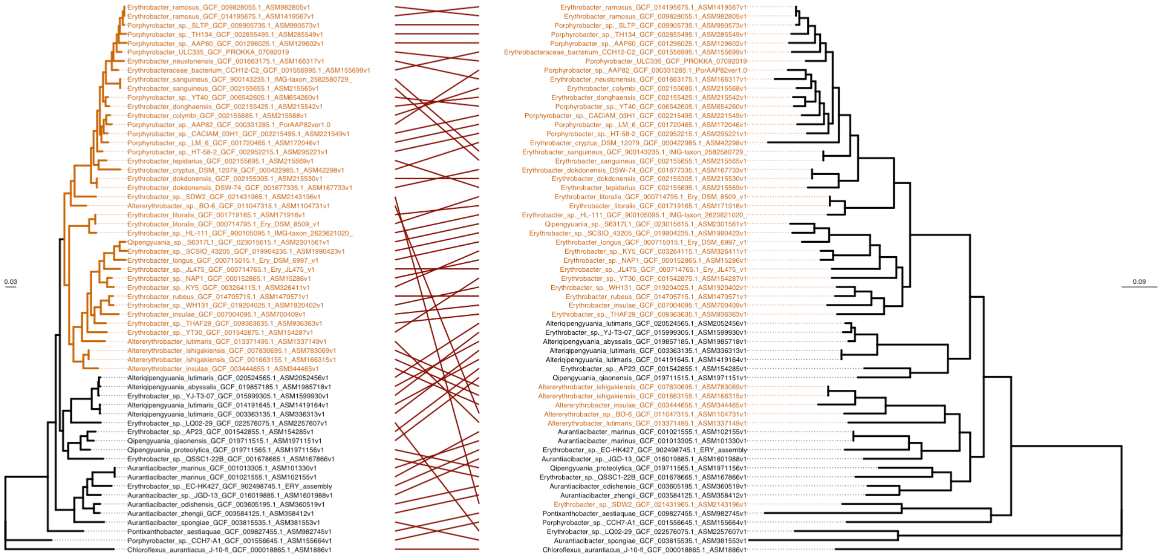

SFigure 5: The anoxygenic phototrophy superoperon is primarily transferred vertically in Erythrobacteraceae. In general, the branching order is congruent between a tree made from concatenated conserved core-genome proteins (left) and a tree of concatenated proteins belonging to the anoxygenic phototrophy operon (right). Exceptions are most likely due to horizontal gene transfers. Scale bar: average number of substitutions per site.

A.

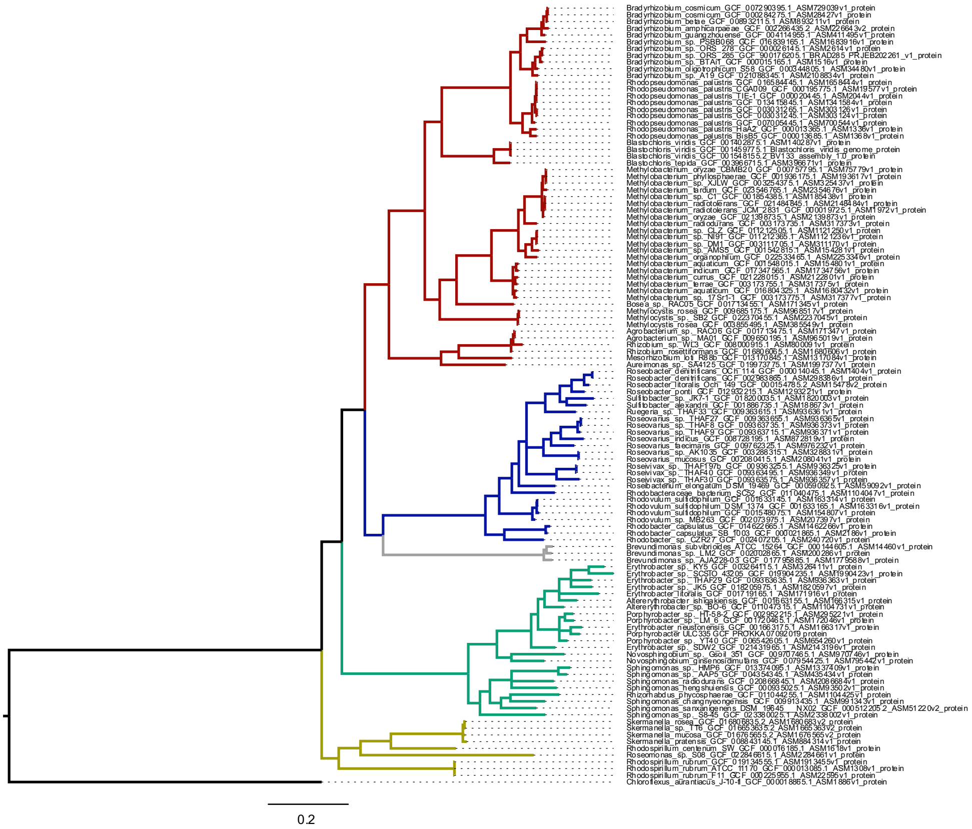
Bradyrhizobium_cosmicum_GCF_007290395.1_ASM729039v1_protein
Bradyrhizobium_cosmicum_GCF_000284275.1_ASM28427v1_protein
Bradyrhizobium_betae_GCF_008932115.1_ASM893211v1_protein
Bradyrhizobium_amphicarpaeae_GCF_002266435.2_ASM226643v2_protein
Bradyrhizobium_guangzhouense_GCF_004114955.1_ASM411495v1_protein
Bradyrhizobium_sp._PSBB068_GCF_016839165.1_ASM1683916v1_protein
Bradyrhizobium_sp._ORS_278_GCF_000026145.1_ASM2614v1_protein
Bradyrhizobium_sp._ORS_285_GCF_900176205.1_BRAD285_PRJEB202261_v1_protein
Bradyrhizobium_sp._BTAi1_GCF_000015165.1_ASM1516v1_protein
Bradyrhizobium_oligotrophicum_S58_GCF_000344805.1_ASM34480v1_protein
Bradyrhizobium_sp._A19_GCF_021088345.1_ASM2108834v1_protein
Rhodopseudomonas_palustris_GCF_016584445.1_ASM1658444v1_protein
Rhodopseudomonas_palustris_CGA009_GCF_000195775.1_ASM19577v1_protein
Rhodopseudomonas_palustris_TIE-1_GCF_000020445.1_ASM2044v1_protein
Rhodopseudomonas_palustris_GCF_013415845.1_ASM1341584v1_protein
Rhodopseudomonas_palustris_GCF_003031265.1_ASM303126v1_protein
Rhodopseudomonas_palustris_GCF_003031245.1_ASM303124v1_protein
Rhodopseudomonas_palustris_GCF_007005445.1_ASM700544v1_protein
Rhodopseudomonas_palustris_HaA2_GCF_000013365.1_ASM1336v1_protein
Rhodopseudomonas_palustris_BisB5_GCF_000013685.1_ASM1368v1_protein
Blastochloris_viridis_GCF_001402875.1_ASM140287v1_protein
Blastochloris_viridis_GCF_001459775.1_Blastochloris_viridis_genome_protein
Blastochloris_viridis_GCF_001548155.2_BV133_assembly_1.0_protein
Blastochloris_tepida_GCF_003966715.1_ASM396671v1_protein
Methylobacterium_oryzae_CBMB20_GCF_000757795.1_ASM75779v1_protein
Methylobacterium_phyllosphaerae_GCF_001936175.1_ASM193617v1_protein
Methylobacterium_sp._XJLW_GCF_003254375.1_ASM325437v1_protein
Methylobacterium_tardum_GCF_023546765.1_ASM2354676v1_protein
Methylobacterium_sp._C1_GCF_001854385.1_ASM185438v1_protein
Methylobacterium_radiotolerans_GCF_021484845.1_ASM2148484v1_protein
Methylobacterium_radiotolerans_JCM_2831_GCF_000019725.1_ASM1972v1_protein
Methylobacterium_oryzae_GCF_021398735.1_ASM2139873v1_protein
Methylobacterium_radiodurans_GCF_003173735.1_ASM317373v1_protein
Methylobacterium_sp._CLZ_GCF_011212505.1_ASM1121250v1_protein
Methylobacterium_sp._NI91_GCF_011212365.1_ASM1121236v1_protein
Methylobacterium_sp._DM1_GCF_003111705.1_ASM311170v1_protein
Methylobacterium_sp._AMS5_GCF_001542815.1_ASM154281v1_protein
Methylobacterium_organophilum_GCF_022533465.1_ASM2253346v1_protein
Methylobacterium_aquaticum_GCF_001548015.1_ASM154801v1_protein
Methylobacterium_indicum_GCF_017347565.1_ASM1734756v1_protein
Methylobacterium_currus_GCF_021228015.1_ASM2122801v1_protein
Methylobacterium_terrae_GCF_003173755.1_ASM317375v1_protein
Methylobacterium_aquaticum_GCF_016804325.1_ASM1680432v1_protein
Methylobacterium_sp._17Sr1-1_GCF_003173775.1_ASM317377v1_protein
Bosea_sp._RAC05_GCF_001713455.1_ASM171345v1_protein
Methylocystis_rosea_GCF_009685175.1_ASM968517v1_protein
Methylocystis_sp._SB2_GCF_022370455.1_ASM2237045v1_protein
Methylocystis_rosea_GCF_003855495.1_ASM385549v1_protein
Agrobacterium_sp._RAC06_GCF_001713475.1_ASM171347v1_protein
Agrobacterium_sp._MA01_GCF_009650195.1_ASM965019v1_protein
Rhizobium_sp._WL3_GCF_008000915.1_ASM800091v1_protein
Rhizobium_rosettiformans_GCF_016806065.1_ASM1680606v1_protein
Mesorhizobium_loti_R88b_GCF_013170845.1_ASM1317084v1_protein
Aureimonas_sp._SA4125_GCF_019973775.1_ASM1997377v1_protein
Roseobacter_denitrificans_OCh_114_GCF_000014045.1_ASM1404v1_protein
Roseobacter_denitrificans_GCF_002983865.1_ASM298386v1_protein
Roseobacter_litoralis_Och_149_GCF_000154785.2_ASM15478v2_protein
Roseobacter_ponti_GCF_012932215.1_ASM1293221v1_protein
Sulfitobacter_sp._JK7-1_GCF_018200035.1_ASM1820003v1_protein
Sulfitobacter_alexandrii_GCF_001886735.1_ASM188673v1_protein
Ruegeria_sp._THAF33_GCF_009363615.1_ASM936361v1_protein
Roseovarius_sp._THAF27_GCF_009363655.1_ASM936365v1_protein
Roseovarius_sp._THAF8_GCF_009363735.1_ASM936373v1_protein
Roseovarius_sp._THAF9_GCF_009363715.1_ASM936371v1_protein
Roseovarius_indicus_GCF_008728195.1_ASM872819v1_protein
Roseovarius_faecimaris_GCF_009762325.1_ASM976232v1_protein
Roseovarius_sp._AK1035_GCF_003288315.1_ASM328831v1_protein
Roseovarius_mucosus_GCF_002080415.1_ASM208041v1_protein
Roseivivax_sp._THAF197b_GCF_009363255.1_ASM936325v1_protein
Roseivivax_sp._THAF40_GCF_009363495.1_ASM936349v1_protein
Roseivivax_sp._THAF30_GCF_009363575.1_ASM936357v1_protein
Roseibacterium_elongatum_DSM_19469_GCF_000590925.1_ASM59092v1_protein
Rhodobacteraceae_bacterium_SC52_GCF_011040475.1_ASM1104047v1_protein
Rhodovulum_sulfidophilum_GCF_001633145.1_ASM163314v1_protein
Rhodovulum_sulfidophilum_DSM_1374_GCF_001633165.1_ASM163316v1_protein
Rhodovulum_sulfidophilum_GCF_001548075.1_ASM154807v1_protein
Rhodovulum_sp._MB263_GCF_002073975.1_ASM207397v1_protein
Rhodobacter_capsulatus_GCF_014622665.1_ASM1462266v1_protein
Rhodobacter_capsulatus_SB_1003_GCF_000021865.1_ASM2186v1_protein
Rhodobacter_sp._CZR27_GCF_002407205.1_ASM240720v1_protein
Brevundimonas_subvibrioides_ATCC_15264_GCF_000144605.1_ASM14460v1_protein
Brevundimonas_sp._LM2_GCF_002002865.1_ASM200286v1_protein
Brevundimonas_sp._AJA228-03_GCF_017795885.1_ASM1779588v1_protein
Erythrobacter_sp._KY5_GCF_003264115.1_ASM326411v1_protein
Erythrobacter_sp._SCSIO_43205_GCF_019904235.1_ASM1990423v1_protein
Erythrobacter_sp._THAF29_GCF_009363635.1_ASM936363v1_protein
Erythrobacter_sp._JK5_GCF_018205975.1_ASM1820597v1_protein
Erythrobacter_litoralis_GCF_001719165.1_ASM171916v1_protein
Altererythrobacter_ishigakiensis_GCF_001663155.1_ASM166315v1_protein
Altererythrobacter_sp._BO-6_GCF_011047315.1_ASM1104731v1_protein
Porphyrobacter_sp._HT-58-2_GCF_002952215.1_ASM295221v1_protein
Porphyrobacter_sp._LM_6_GCF_001720465.1_ASM172046v1_protein
Erythrobacter_neustonensis_GCF_001663175.1_ASM166317v1_protein
Porphyrobacter ULC335 GCF PROKKA 07092019 protein
Porphyrobacter_sp._YT40_GCF_006542605.1_ASM654260v1_protein
Erythrobacter_sp._SDW2_GCF_021431965.1_ASM2143196v1_protein
Novosphingobium_sp._Gsoil_351_GCF_009707465.1_ASM970746v1_protein
Novosphingobium_ginsenosidimutans_GCF_007954425.1_ASM795442v1_protein
Sphingomonas_sp._HMP6_GCF_013374095.1_ASM1337409v1_protein
Sphingomonas_sp._AAP5_GCF_004354345.1_ASM435434v1_protein
Sphingomonas_radiodurans_GCF_020866845.1_ASM2086684v1_protein
Sphingomonas_hengshuiensis_GCF_000935025.1_ASM93502v1_protein
Rhizorhabdus_phycosphaerae_GCF_011044255.1_ASM1104425v1_protein
Sphingomonas_changnyeongensis_GCF_009913435.1_ASM991343v1_protein
Sphingomonas_sanxanigenens_DSM_19645___NX02_GCF_000512205.2_ASM51220v2_protein
Sphingomonas_sp._S8-45_GCF_023380025.1_ASM2338002v1_protein
Skermanella_rosea_GCF_016806835.2_ASM1680683v2_protein
Skermanella_sp._TT6_GCF_016653635.2_ASM1665363v2_protein
Skermanella_mucosa_GCF_016765655.2_ASM1676565v2_protein
Skermanella_pratensis_GCF_008843145.1_ASM884314v1_protein
Rhodospirillum_centenum_SW_GCF_000016185.1_ASM1618v1_protein
Roseomonas_sp._S08_GCF_022846615.1_ASM2284661v1_protein
Rhodospirillum_rubrum_GCF_019134555.1_ASM1913455v1_protein
Rhodospirillum_rubrum_ATCC_11170_GCF_000013085.1_ASM1308v1_protein
Rhodospirillum_rubrum_F11_GCF_000225955.1_ASM22595v1_protein
Chloroflexus_aurantiacus_J-10-fl_GCF_000018865.1_ASM1886v1_protein
0.2

B.

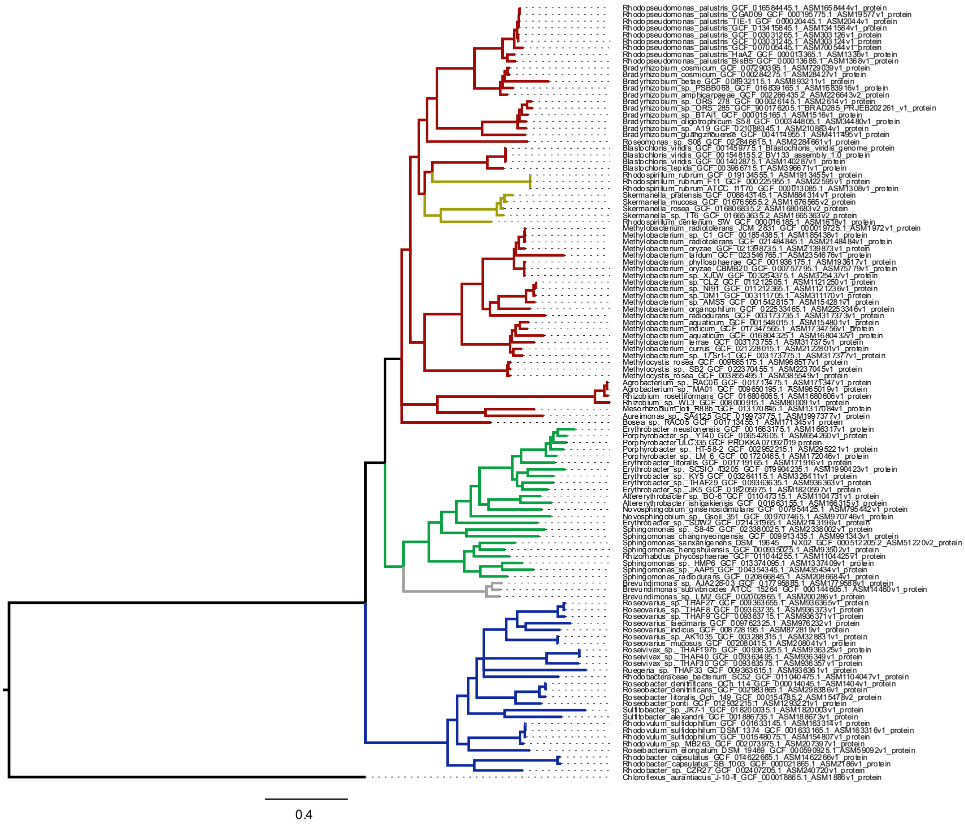

SFigure 6: The anoxygenic phototrophy superoperon is primarily transferred vertically in alphaproteobacteria from the class level down. In general, the branching order is congruent, within classes, between a tree made from concatenated conserved core-genome proteins (A) and a tree of concatenated proteins belonging to the anoxygenic phototrophy operon (B). Exceptions are most likely due to horizontal gene transfers. Yellow: Rhodospirillales; green: Sphingomonadales; grey: Caulobacterales; blue: Rhodobacterales; red: Rhizobiales. Scale bar: average number of substitutions per site.

A.

Total Sequence Length (bp):	3 618 402
Number of Sequences:	1
GC content (%):	64.6
Number of coding sequences:	3 443
Average Protein Length:	321.1
Coding Ratio (%):	91.4
Number of CDS:	3 396
Number of rRNAs:	3
Number of tRNAs:	44
Number of CRISPRs:	0

B.

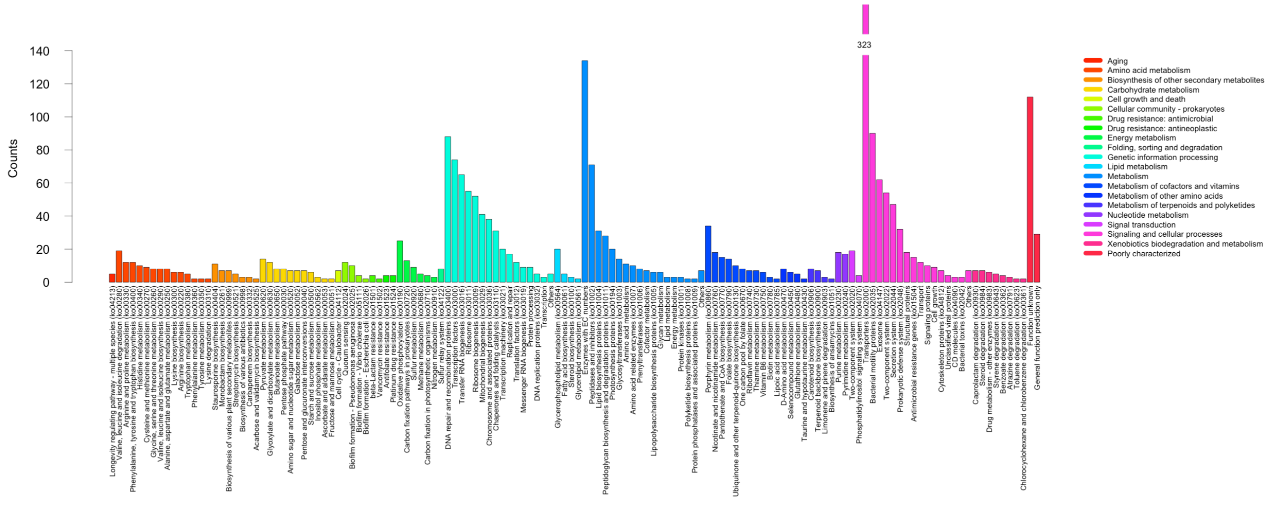

C.

Gene	HMM_Models	Evalues	Description
KVF90_01410	BLUF	1.80E-25	BLUF domain at N-terminus; the rest of the protein does not give a hit in NCBI conserved domain database
KVF90_02750	BLUF	1.40E-24	BLUF domain at N-terminus making up more than 50% of the protein
KVF90_02755	BLUF	5.00E-31	BLUF domain at N-terminus making up more than 50% of the protein
KVF90_05705	PHY	7.20E-41	Bacteriophytochrome domain at N-terminus followed with a phosphoacceptor receiver (REC) domain
KVF90_08400	BLUF	8.70E-27	BLUF domain at N-terminus making up more than 50% of the protein
KVF90_16685	BLUF	1.40E-26	BLUF domain at N-terminus making up more than 50% of the protein

SFigure 7: Global statistics and KEGG analysis of the genome of *Porphyrobacter* sp. ULC335. A. General characteristics of the genome of *Porphyrobacter* sp. ULC335. B. Functional classification of proteins encoded in the genome based on KEGG categories (two-levels). C. Table of all *Porphyrobacter* sp. ULC335 proteins containing light sensing domains (blue-sensing LOV or BLUF domains, or red-sensing bacteriophytochrome).

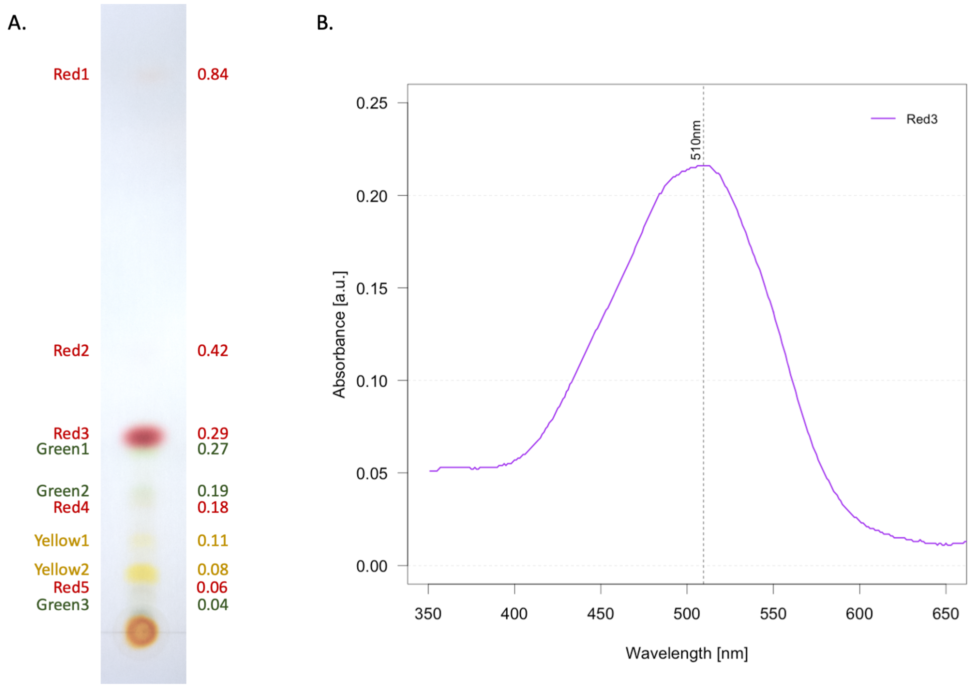

SFigure 8: *Porphyrobacter* sp. ULC335 produces a range of carotenoids and bacteriochlorophyll *a* derivatives. A. Thin Layer Chromatography of pigments extracted from *Porphyrobacter* sp. ULC335. Red and yellow pigments are most likely carotenoids, green pigments precursors or derivatives of bacteriochlorophyll *a.* B. Absorbance spectrum of the main red pigment ("Red3") eluted from the TLC plate. The UV-visible absorbance spectrum of the main purple-red carotenoid is indicative of bacteriorubixanthinal, found in *Erythrobacter longus* and close relatives as the main reaction center-bound carotenoid[1–3].

A.

Protein	Locus in *Por.* sp. ULC335	blastp e-value	Function
Mch	KVF90_00360	3.19E-13	β-methylmalyl-CoA dehydratase
SmtB	KVF90_00365	5.89E-32	Succinyl-CoA:(S)-malyl-CoA transferase, subunit B
Meh	KVF90_03630	1.37E-51	Mesaconyl-C4-CoA hydratase
Mct	KVF90_03640	3.66E-90	Mesaconyl-C1-CoA-C4-CoA transferase
AccA	KVF90_08270	1.18E-81	Acetyl-Coenzyme-A carboxylase, subunit A
Pcs	KVF90_08260	0.00E+00	Propionyl-CoA synthase
Mcr	KVF90_08265	0.00E+00	Malonyl-CoA reductase
Mcl	KVF90_09475	8.58E-21	(S)-malyl-CoA/β-methylmalyl-CoA/(S)-citramalyl-CoA (MMC) lyase

B.

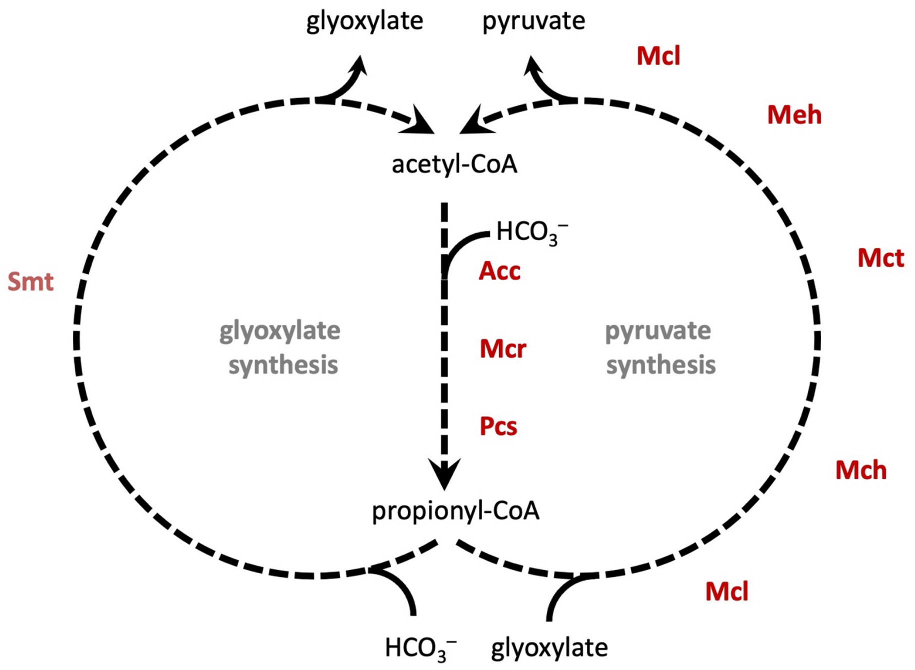

SFigure 9: Homologs of several *Chloroflexus aurantiacus* enzymes participating to the 3hydroxypropionate bicycle for carbon fixation are found in *Porphyrobacter* sp. ULC335 genome. A. Table of enzymes found in the *Porphyrobacter* sp. ULC335

genome. The AccA protein is not considered a signature protein, but is often found associated with Pcs and Mcr homologs in genomes encoding the full 3-hydroxypropionate bicycle. The Mcl and the SmtB have relatively high e-values; an SmtA homolog is missing; the pathway is therefore considered incomplete like the one found in *Erythrobacter* sp. NAP1[4]. B. Schematic representation of the 3¬hydroxypropionate bicycle with the enzymes found the *Porphyrobacter* sp. ULC335 genome indicated in red.

A.

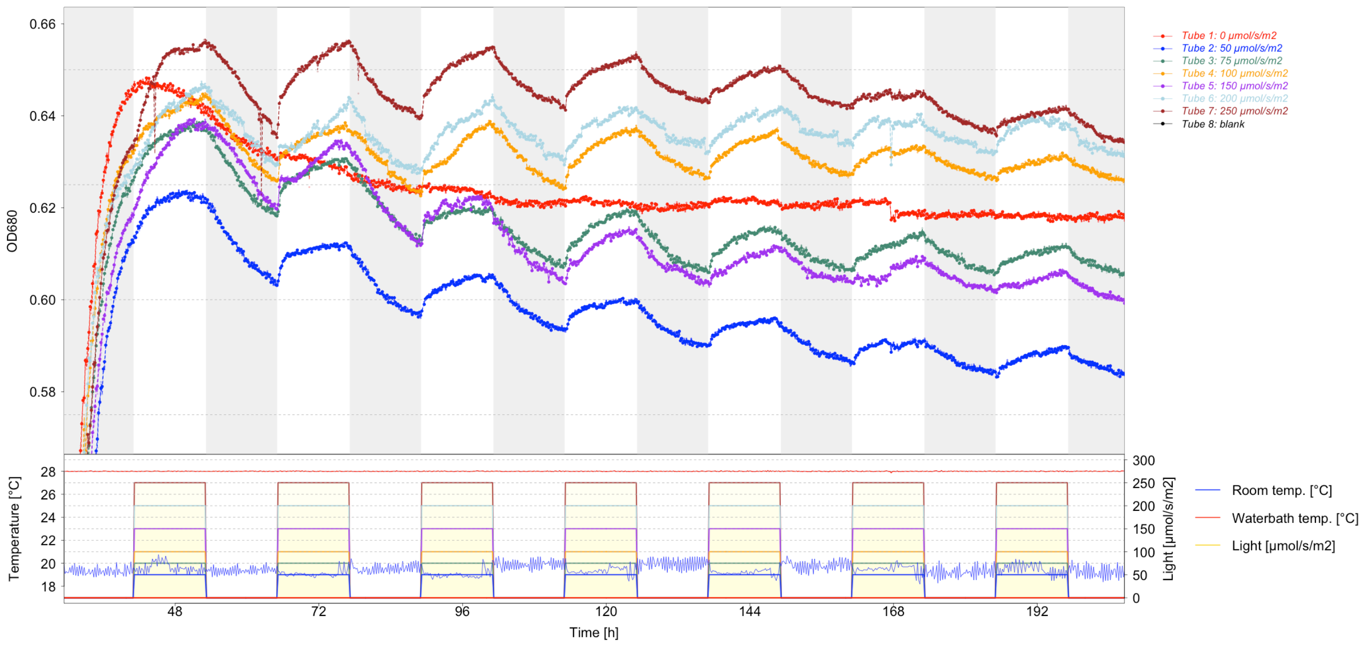

B.

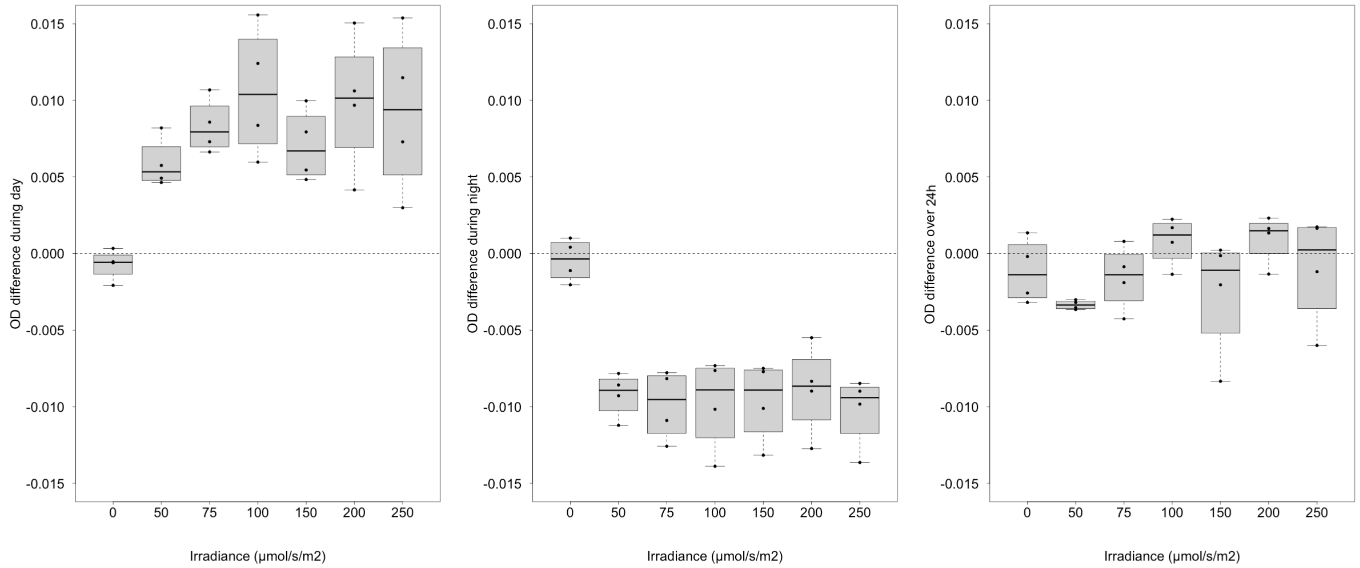

SFigure 10: Growth curves of *Porphyrobacter* sp. ULC335 under 12:12 dark-light alternance in a range of light intensities. A. Rhythms are observed from 50 µmol/s/m2 to 250 µmol/s/m2; the maximal optical density tends to increase with light intensity. Growth curve above and conditions (light regime and water bath temperature; room temperature has no impact on culture conditions, but is added as a control) below. B. Boxplot showing the optical density differences (over the day, over

the night and the net difference over 24 hours). The differences were calculated over four full cycles starting at 89h. The increase in density during the day correlates with the light intensity up to 100 µmol/s/m^2, where it saturates; the decrease in density during the night seems dependent on light exposure but independent on light intensity; the net difference is close to zero except for the 50 µmol/s/m^2 treatment, where the detrimental effect of light exposure observed during the night is not compensated by growth during the day.

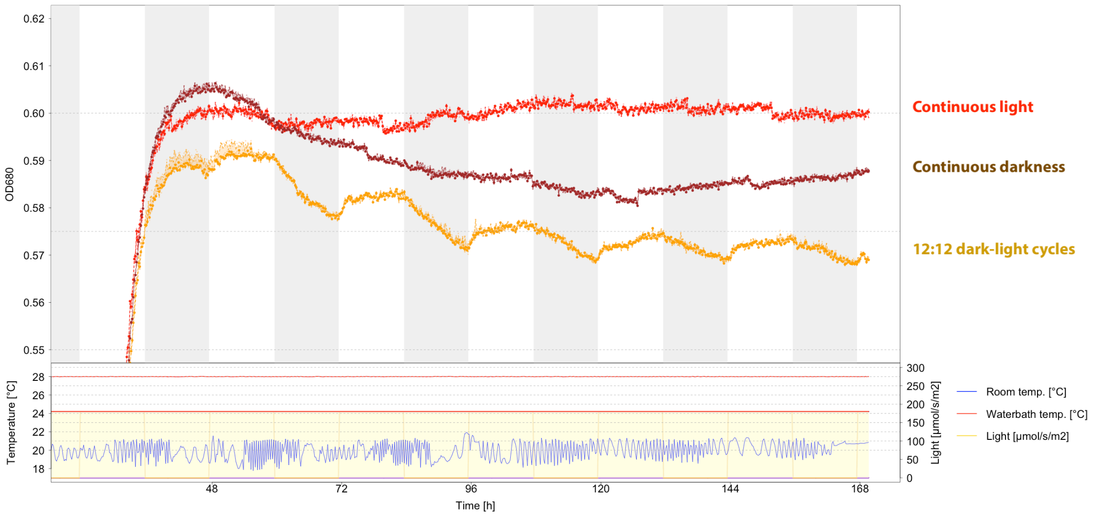

SFigure 11: Growth curves of *Porphyrobacter* sp. ULC335 under continuous light (red), continuous darkness (dark brown), and 12:12 dark-light alternance (yellow), cultured in parallel. Rhythms are only observed under 12:12 dark-light alternance; the optical density under continuous light is consistently higher than under continuous darkness. Growth curve above and conditions (light regime and waterbath temperature; room temperature has no impact on culture conditions, but is added as a control) below.

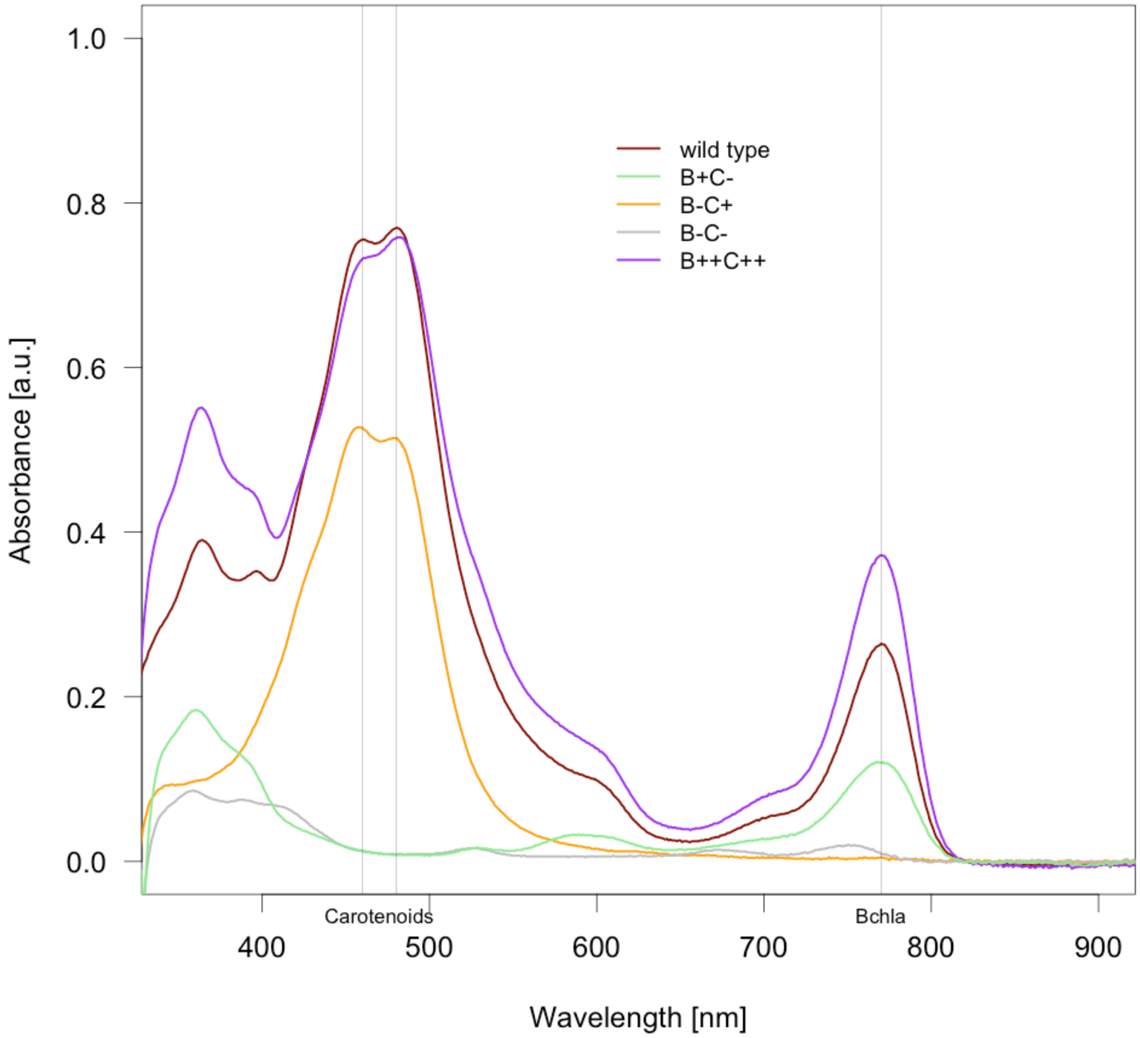

SFigure 12: Absorbance spectra of *Porphyrobacter* sp. ULC335 and its four transposon mutants. The figure shows the absorbance profile of 7:2 acetone-methanol extracts of 72-hour batch cultures of *Porphyrobacter* sp. ULC335 and four mutants: the carotenoid mutant $B^{+}C^{-}$, the bacteriochlorophyll *a*-null mutant $B^{-}C^{+}$, which produces less carotenoids as well, the carotenoid-bacteriochlorophyll *a*-null mutant $B^{-}C^{-}$, and the carotenoid-bacteriochlorophyll *a* overproducer $B^{++}C^{++}$. Bacteria were grown in the dark at 30°C.

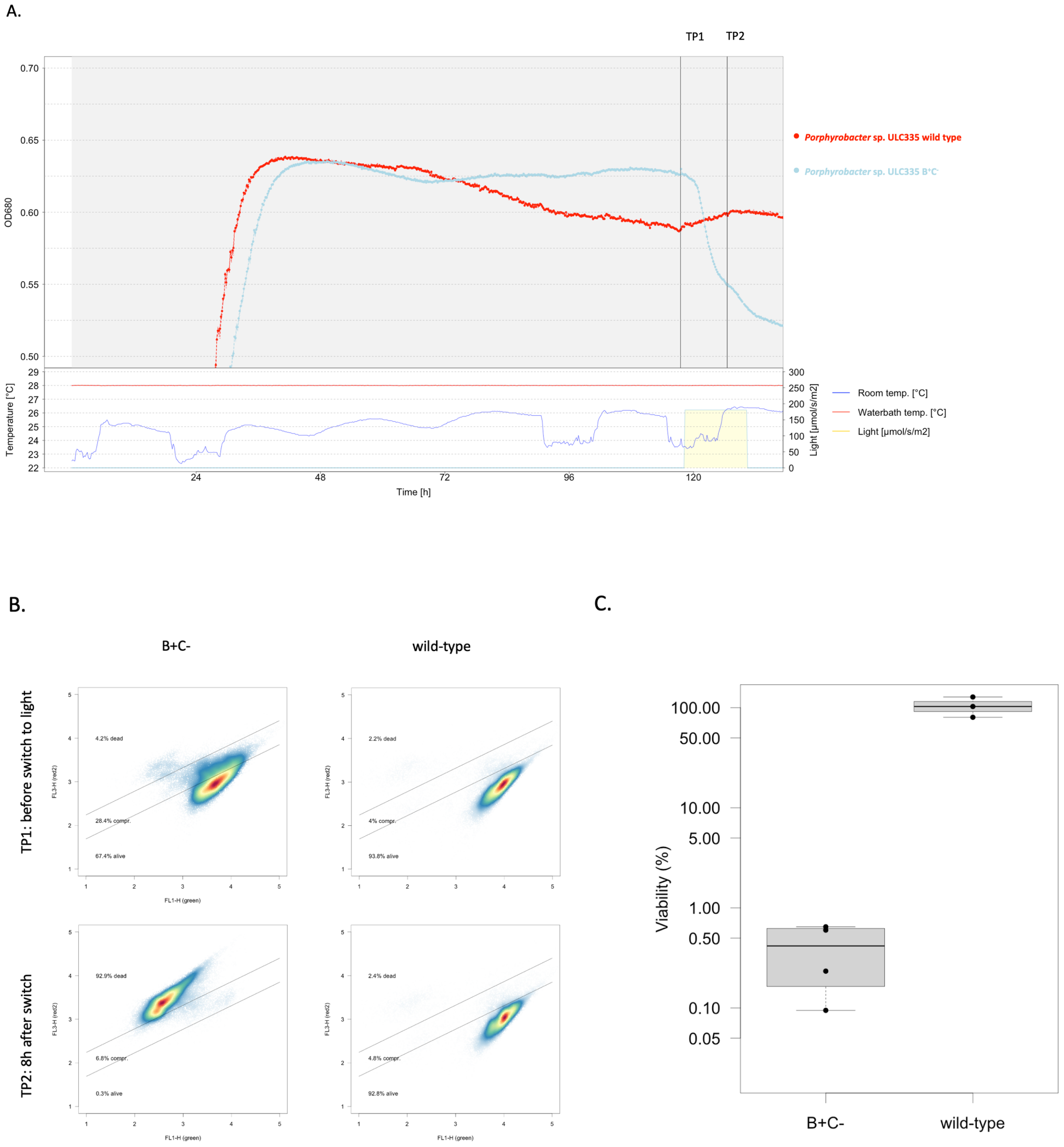

SFigure 13: Upon light exposure strain B+C- loses viability. A. Cultures of *Porphyrobacter* sp. ULC335 wild type and the carotenoid mutant B+C- were grown in the dark for five days and then exposed to light (180 µmol/s/m2) for twelve hours. The growth curve shows a clear decrease in optical density right after exposure to light in the carotenoid mutant. B. The cultures were tested for viability right before and

8 hours after the switch to light using Life/Dead staining. The density graphs show that light induces cell permeabilization (death) in the B+C- strain but not in the wild type strain under the same conditions. C. Colony-forming units [CFUs] confirm that the viability of the B+C- strain decreases by more than two orders of magnitude when the cultures are exposed to light while the viability of the wild type strain remains stable. The viability is the ratio of CFUs at TP1 divided by the CFUs at TP2 for each culture, expressed in percent.

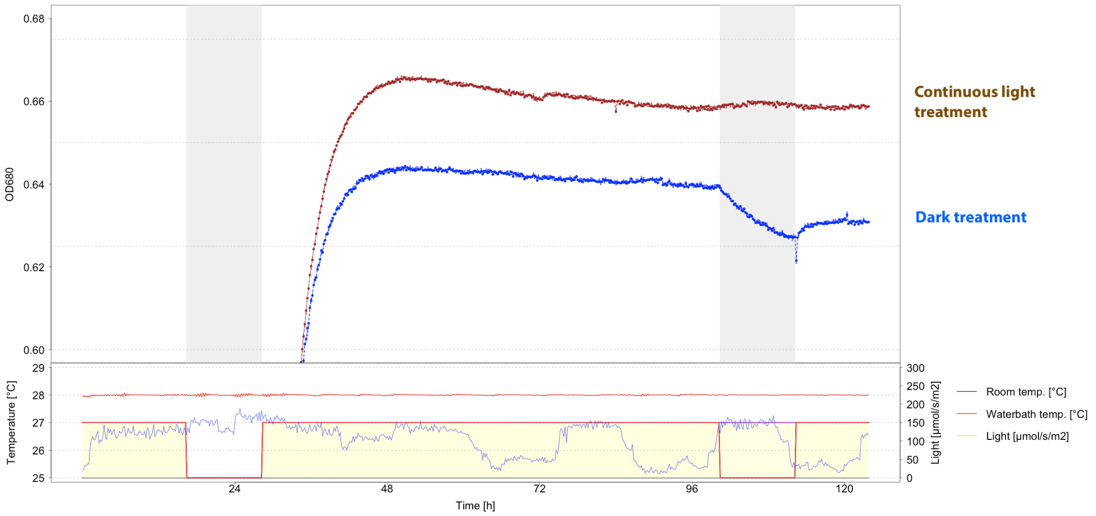

SFigure 14: Actual data on which experimental design figure 3A is based. Growth curve above and conditions (light regime and waterbath temperature; room temperature is only indicative) below. The brown line is from a culture which has been maintained under continuous light as of stationary phase; the blue line is from a culture which has been switched to darkness at approximately 98 hours and switched back to light 12 hours after.

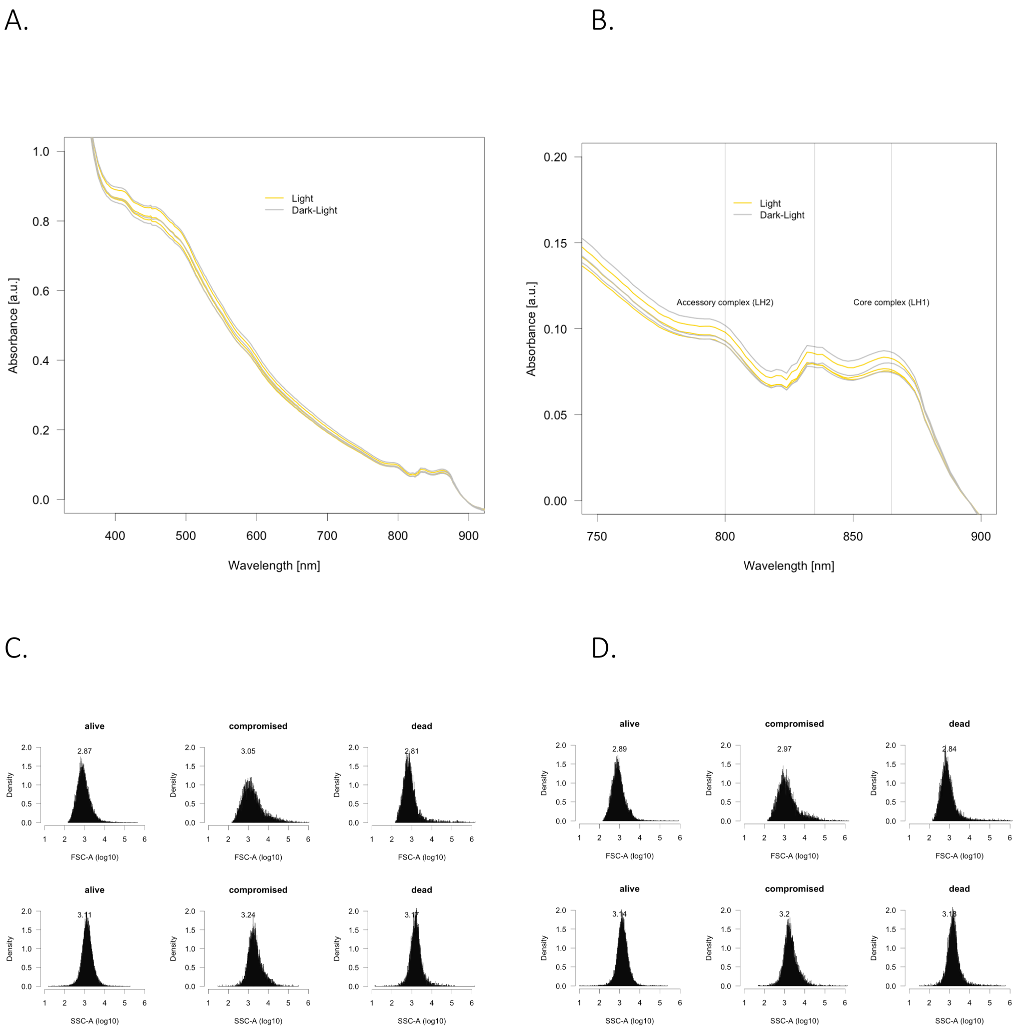

SFigure 15: There is no difference in pigment content, cell size or cell aggregation between cells collected during a light or a dark phase. A. and B. Whole-cell absorbance spectra of five-fold concentrated *Porphyrobacter* sp. ULC335 cultures grown under continuous light (“Light”) or right after a 12-hour exposure to darkness (“Dark-Light”). A. Spectra from 350 to 900 nm showing carotenoid peaks between 450 and 520 nm and the light harvesting complexes between 750 and 900 nm. B. Detailed spectra from 750 to 900 nm showing the peaks corresponding to light harvesting complexes LH1 (800 nm) and LH2 (865 nm). The peak at 835 nm is atypical but could correspond to a secondary peak of the accessory complex (observed around 850nm

in the anaerobic anoxygenic phototroph *Rhodobacter*)[5]. C. and D. Cell size (FSC-A) and cell granularity (SSC-A) of single cells from *Porphyrobacter* sp. ULC335 cultures grown under continuous light (C) or after a 12-hours exposure to darkness D); the values are ventilated based on their Live/Dead staining status. Neither the maxima nor the distributions show obvious differences between the two treatments suggesting that the cells are not different in size and do not aggregate in different ways in one treatment compared to the other

A

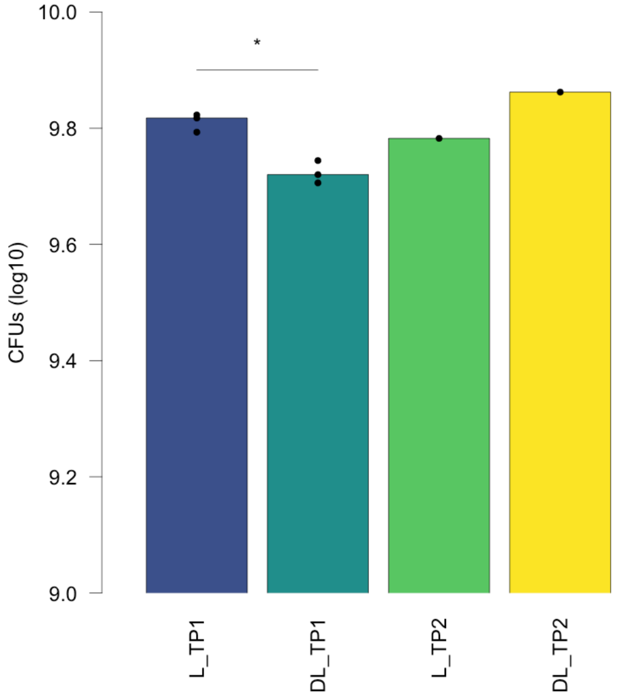

10.0
9.8
9.6
9.4
9.2
9.0
*
CFUs (log10)
L_TP1
DL_TP1
L_TP2
DL_TP2

B.

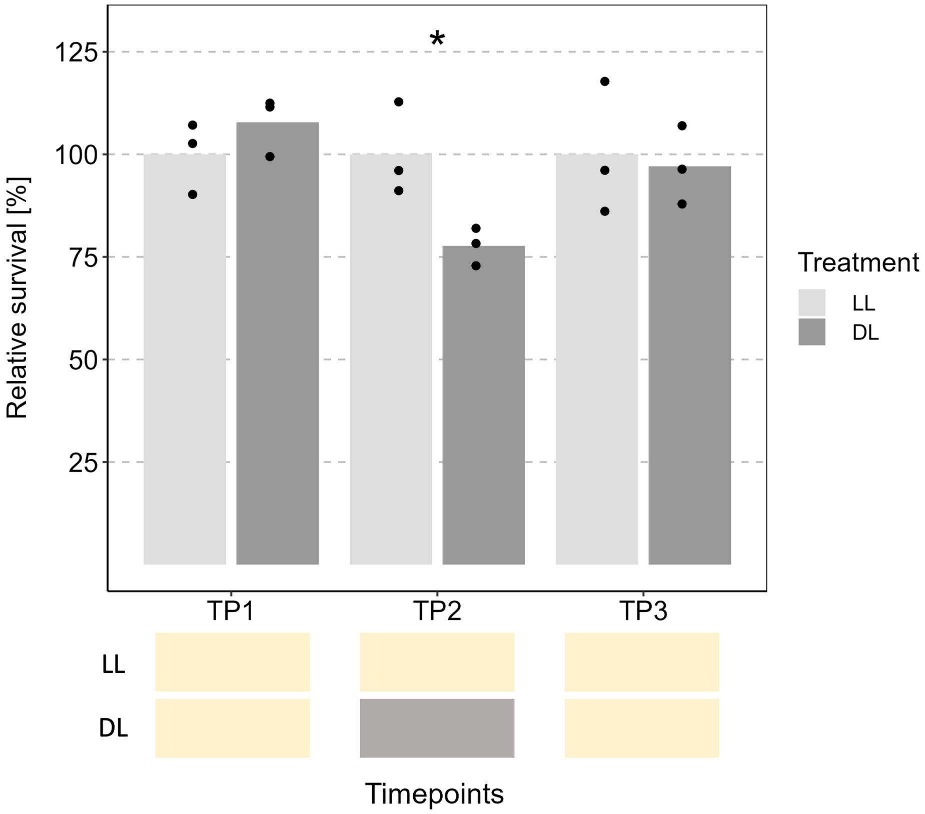

125
100
75
50
25
*
Relative survival [%]
Treatment
LL
DL
TP1
TP2
TP3
LL
DL
Timepoints

SFigure 16: Colony forming units (CFUs) confirm the Live/Dead staining trend. A. This graph shows that the CFUs are significantly lower after an exposure to darkness than under continuous light. Only one sample was taken at the second time point, but it also confirms the recovery after re-exposure to light. The star indicates a statistically significant difference (bilateral Student's t-test, p-value<0.05). B. Results from an independent experiment, which show exactly the same trend. Six *Porphyrobacter* sp. ULC335 independent cultures were grown to stationary phase and maintained under continuous light (150 µmol/s/m^2) for 72 hours; to allow for Bchl*a* accumulation, cultures were exposed to darkness for 12 hours during exponential phase. Then, for three replicates (LDL treatment), light was switched off for 12 hours and switched back on, while the three other tubes were maintained under continuous light (LL treatment). Samples were taken from all six cultures one hour before the switch to darkness, six hours after switch to darkness, and twelve hours after re-exposure to light. In the DL treatment, the viability decreases significantly (paired bilateral Student's t-test, p-value<0.05) during the period of darkness compared to the control and increases back after re-exposure to light. Star: p-value < 0.05.

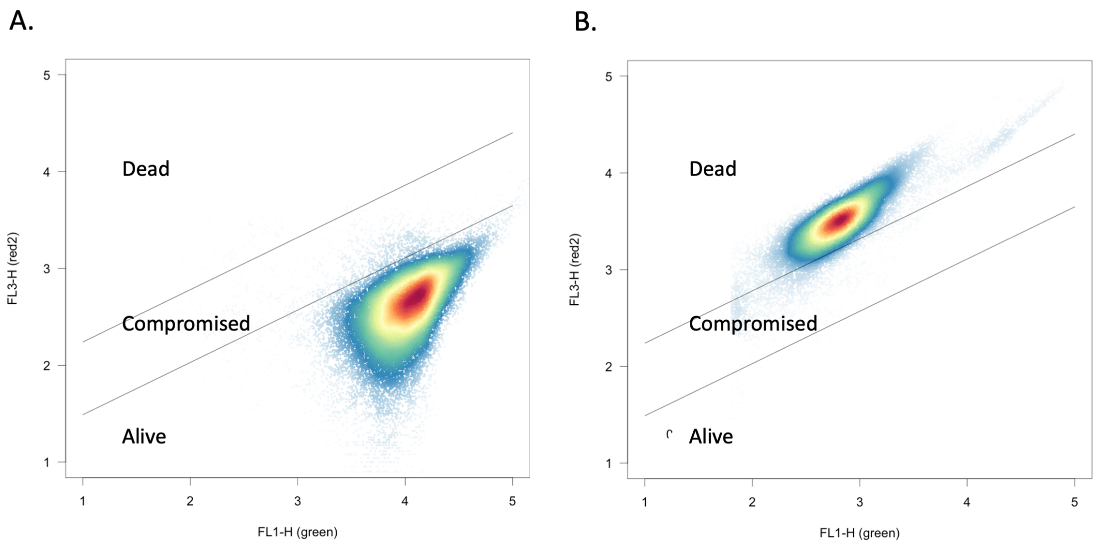

SFigure 17: Distribution of green and red fluorescence values in live and dead *Porphyrobacter* sp. ULC335 cells. A. Live cells from a 36-hour culture show high green (FL-1) and low red signal (FL-3). B. Cells fixed with 2.5% glutaraldehyde show low green and high red signal. The zone in between the lines accommodate the high green and high red signal cells, that we consider “compromised” and in which the “dead dye”, propidium iodide, can enter, but at a slow speed.

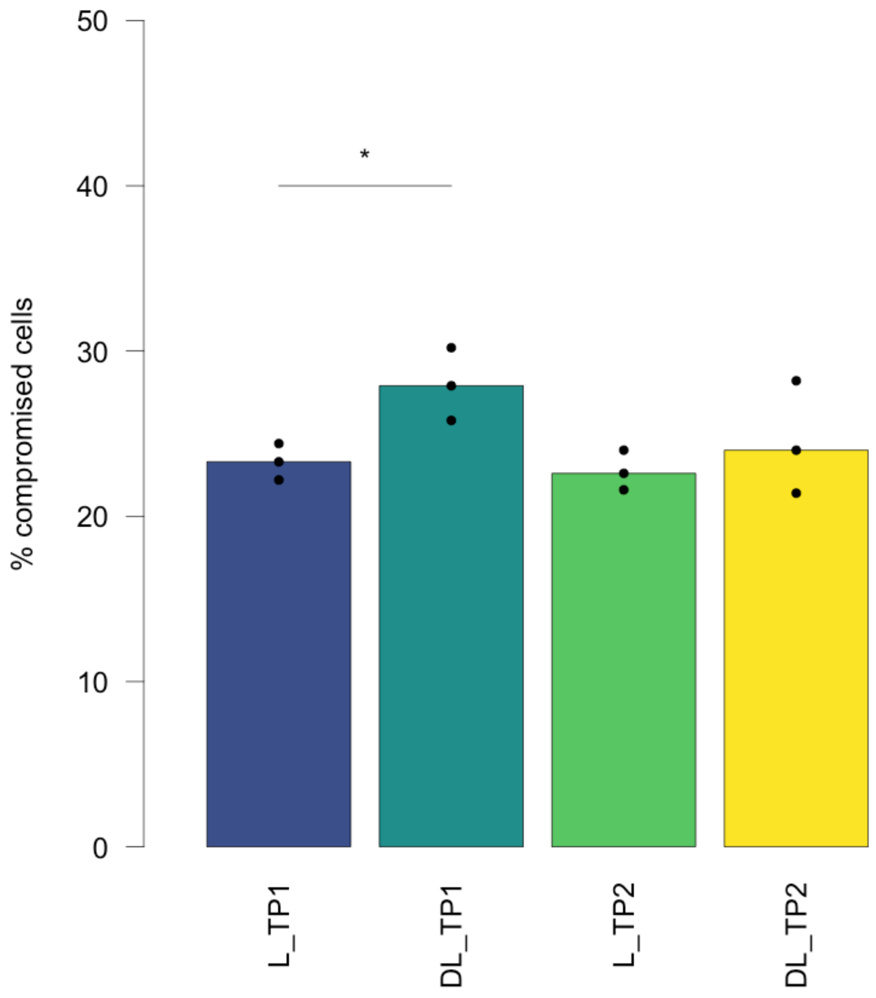

SFigure 18: The proportion of compromised cells is greater in the cultures subjected to a dark phase than in the cultures maintained under continuous light. This graph shows the proportion of cells falling in the "compromised" category, based on flow cytometry, is significantly higher after an exposure to 12-hour darkness than under continuous light. Like for dead cells, the proportions go back to baseline. The star indicates a statistically significant difference (bilateral Student's t-test, p-value<0.05).

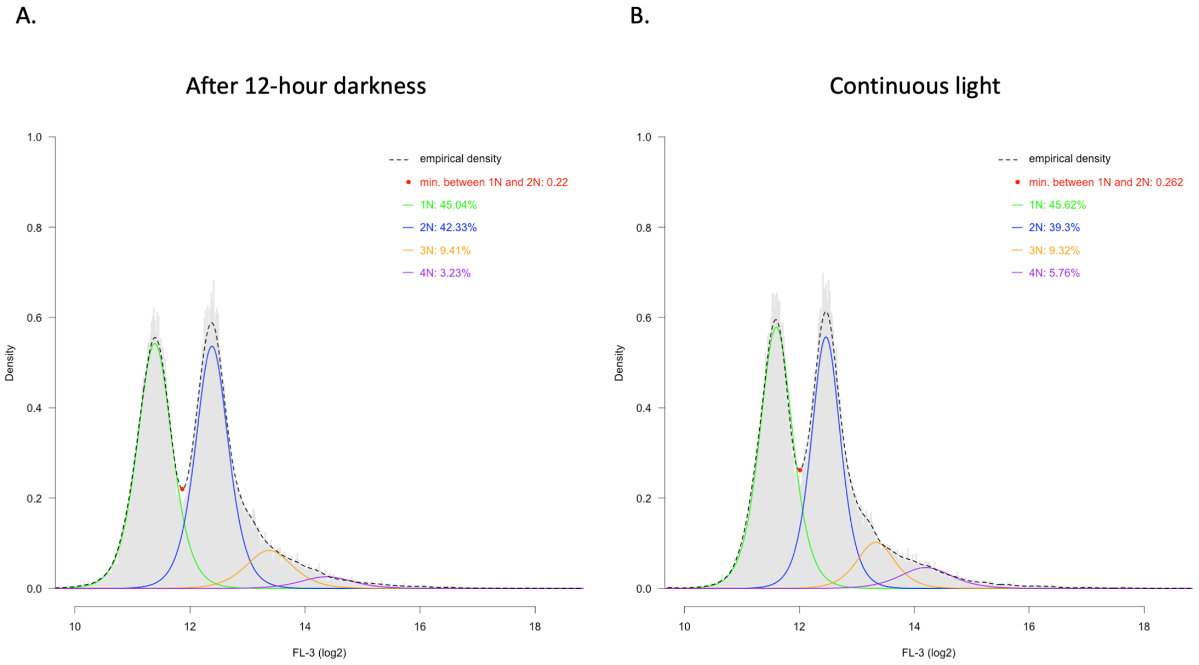

SFigure 19: The replicating-cell population size differs between the dark (A) and continuous light (B) treatments. The graphs show a histogram of the distribution of green fluorescence (FL-1) in DyeCycle Orange-stained cells as measured by flow cytometry. Overlapping the histogram, lines represent the smoothed empirical density over the population (ticked black line), the modelled 1N (green line), 2N (blue line), 3N (orange line), and 4N (purple line) chromosome content peaks as predicted from the location of the 1N and 2N peaks (local maxima and distance between two N values). In both treatments, more than 85% of the cells were likely arrested at 1N-chromosome (before the start of DNA replication) or at 2N chromosomes (after the end of chromosomal replication and before cell division) The relative proportions between the four peaks were not different between treatments. However, the lowest empirical density value between the 1N and the 2N peaks, which is a measure of the

actively replicating population, was significantly lower in cells exposed to the dark than in the cells maintained under continuous light.

A.

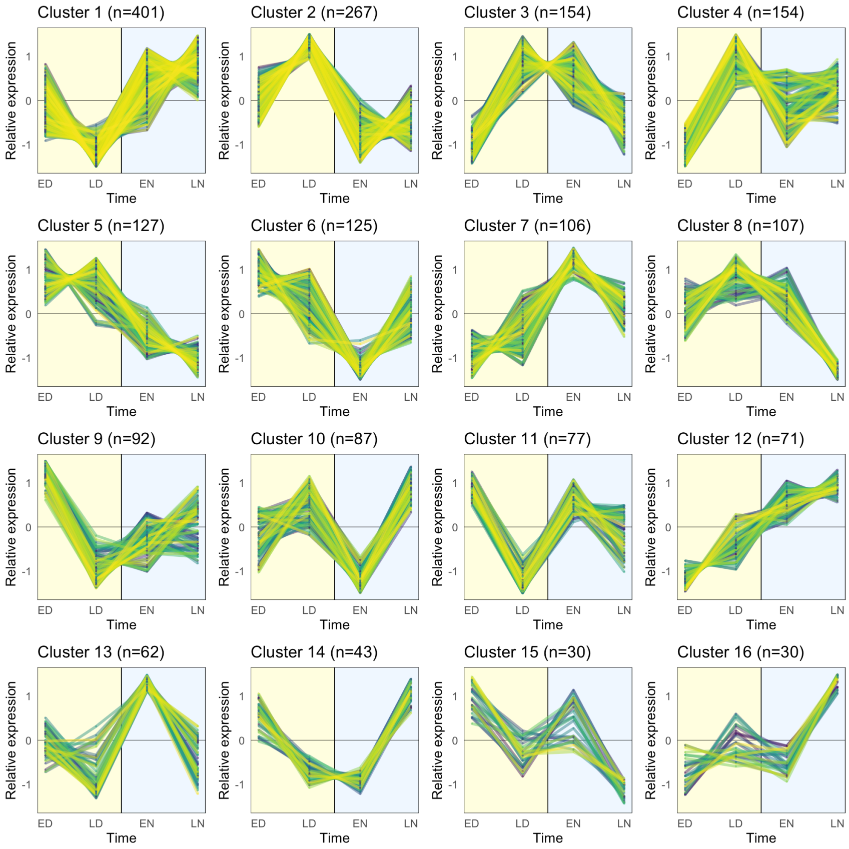

Cluster 1 (n=401)
Cluster 2 (n=267)
Cluster 3 (n=154)
Cluster 4 (n=154)
Cluster 5 (n=127)
Cluster 6 (n=125)
Cluster 7 (n=106)
Cluster 8 (n=107)
Cluster 9 (n=92)
Cluster 10 (n=87)
Cluster 11 (n=77)
Cluster 12 (n=71)
Cluster 13 (n=62)
Cluster 14 (n=43)
Cluster 15 (n=30)
Cluster 16 (n=30)
Relative expression
Time
ED
LD
EN
LN

B.

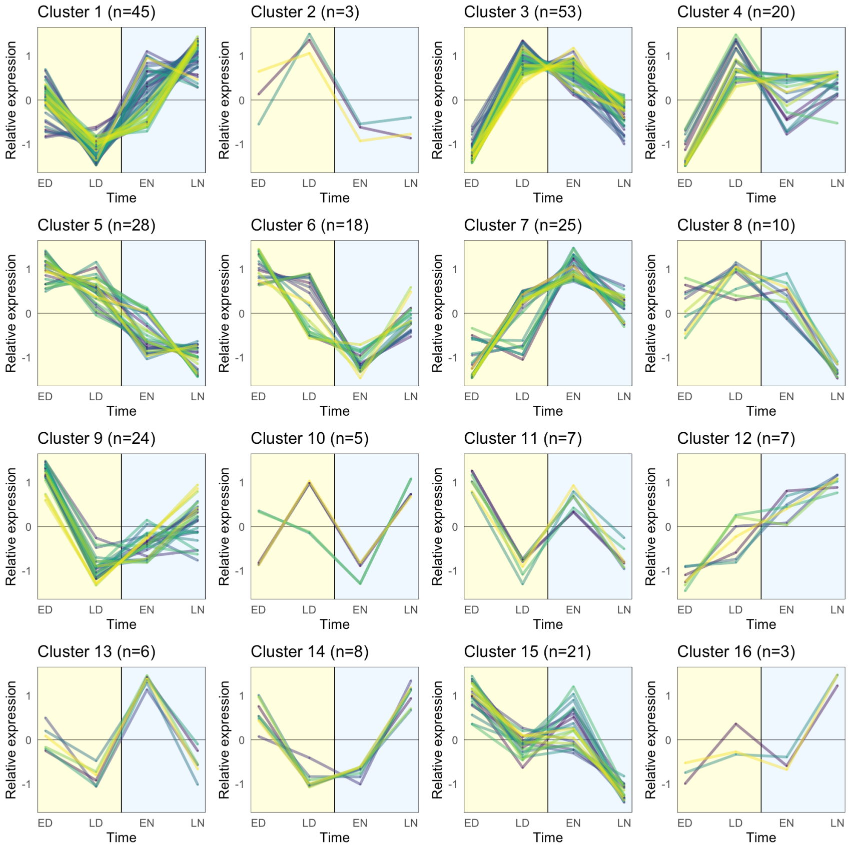

Cluster 1 (n=45)
Cluster 2 (n=3)
Cluster 3 (n=53)
Cluster 4 (n=20)
Cluster 5 (n=28)
Cluster 6 (n=18)
Cluster 7 (n=25)
Cluster 8 (n=10)
Cluster 9 (n=24)
Cluster 10 (n=5)
Cluster 11 (n=7)
Cluster 12 (n=7)
Cluster 13 (n=6)
Cluster 14 (n=8)
Cluster 15 (n=21)
Cluster 16 (n=3)
Relative expression
Time
ED
LD
EN
LN

C.

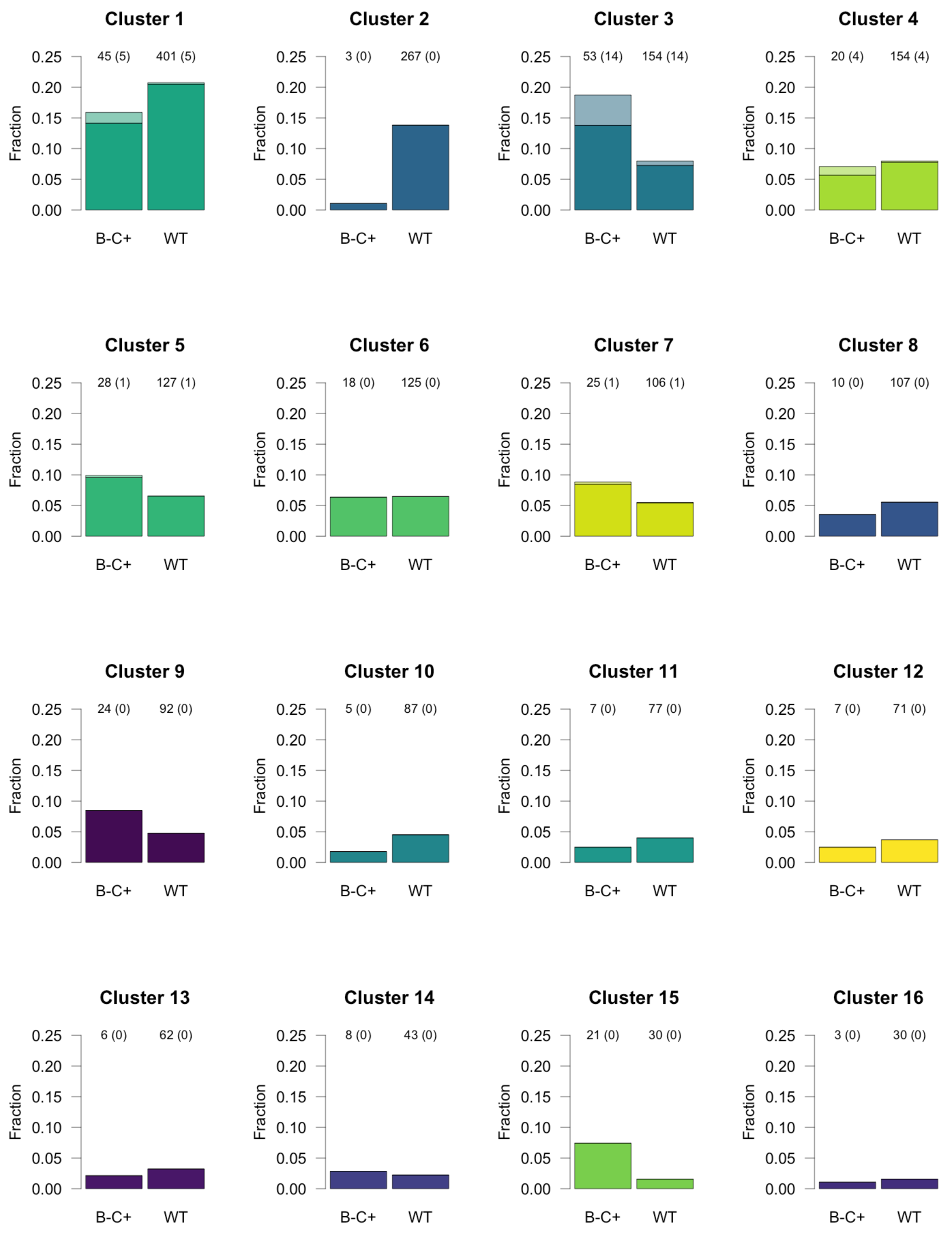

SFigure 20: Clusters of temporally regulated genes in the wild type (A) and the B$^-$C$^+$ mutant (B), and their representation in each strain (C). The figure summarizes information about the temporal regulation of genes in the wild type strain and its Bchl*a* null mutant. A. and B. The clusters of genes showing a consistent light-response pattern in either the wild type (A) or the photosynthesis null mutant B$^-$C$^+$ (B) are presented based on their temporal profile. The datapoints represent the relative transcription rates, i.e. the Z-scores of the rlog-transformed count data. C. The

barplots shows the fraction each cluster represents among temporally regulated genes in either (dark colors) or both (light colors) strains.

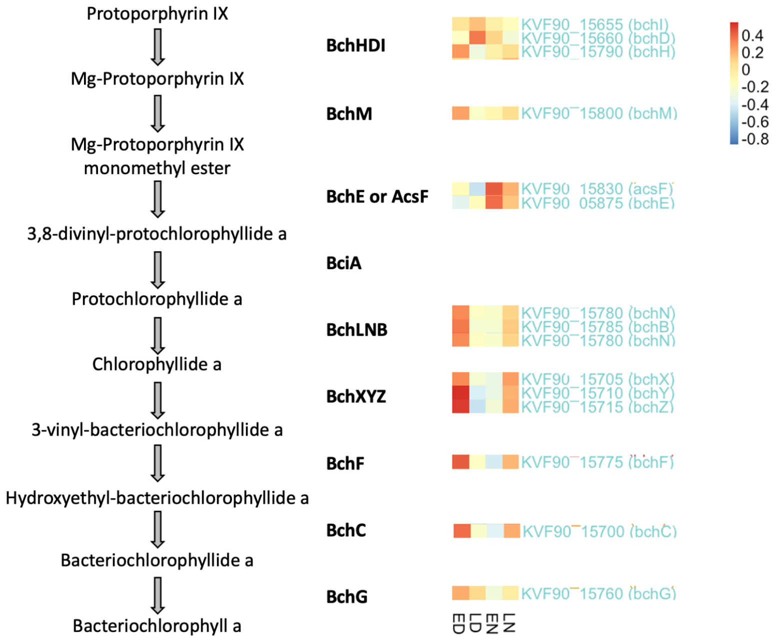

SFigure 21: Schematic representation of the bacteriochlorophyll *a* biosynthesis pathway together with the enzymes involved and their transcriptional profile in the wild type strain. The heatmaps have been normalized to the wild type strain. Three temporal profiles emerge: 1. *bchDI,* maximally expressed at LD, 2. *bchHM, bchLNB, bchXYZ, bchF* and *bchC*, expressed at LN and ED, 3. *bchE* and *acsF*, expressed at EN and LN. EN: early night; LN: late night; ED: early day; LD: late day.

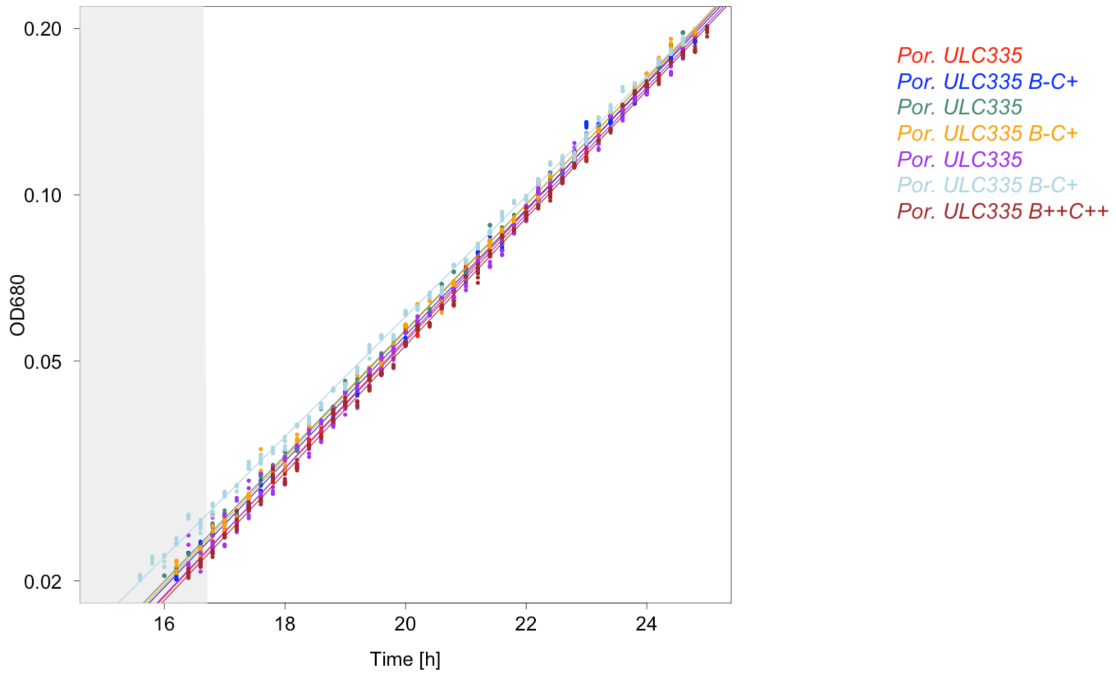

SFigure 22: The growth rates of the wild type, the Bchl*a*-null B$^-$C$^+$ and the Bchl*a*-carotenoid overproducer B^{++}C++ strains do not differ significantly. The figure represents the OD680 as a function of time of three wild type, three B$^-$C$^+$, and one B^{++}C^{++} replicates in early exponential phase. The slope does not significantly differ between the cultures, indicating that the growth rate of the three strains in exponential phase is very similar.

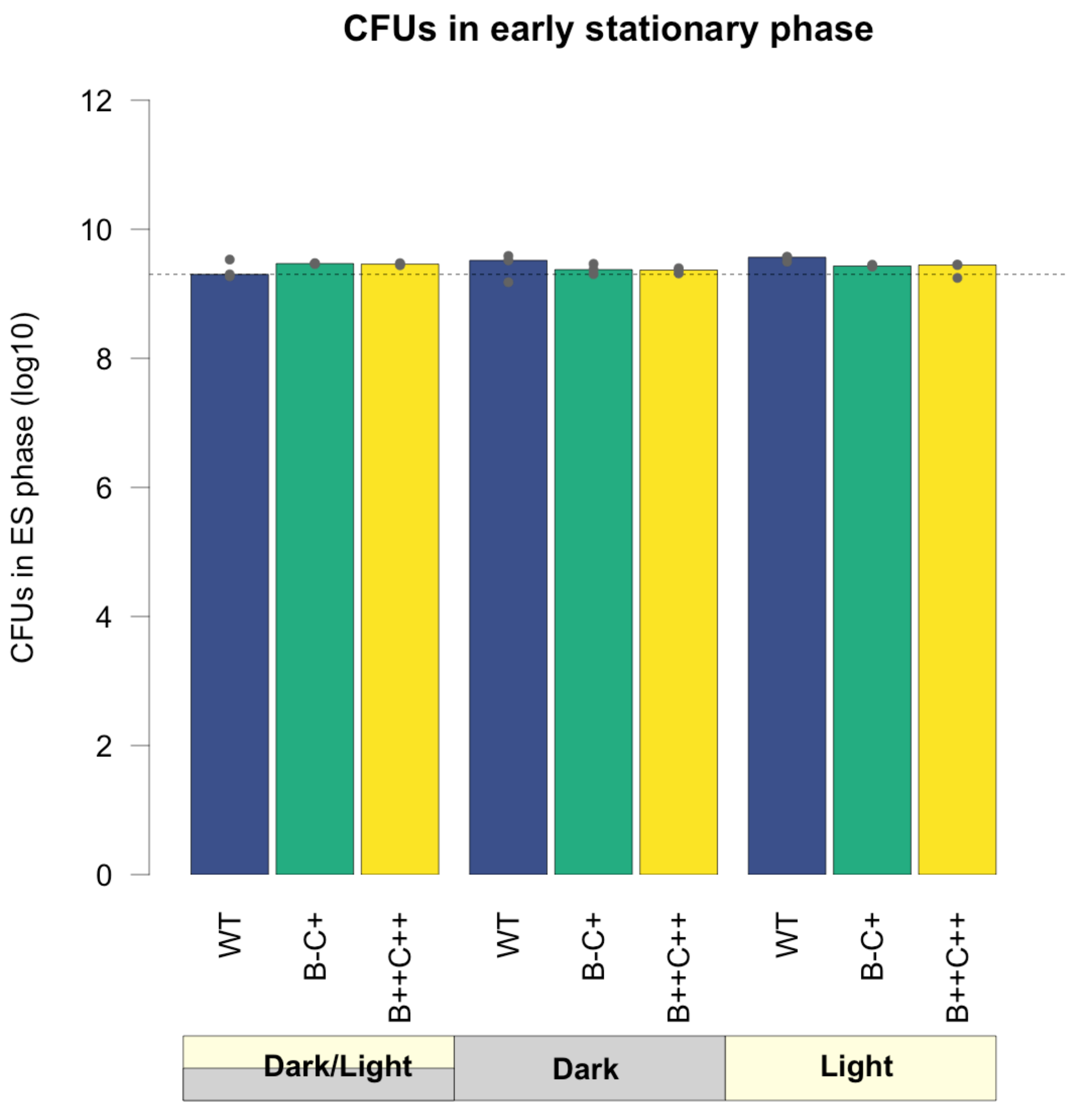

SFigure 23: The colony forming units (CFUs) of the three strains do not differ significantly in early stationary (ES) phase (after 48 hours), whatever the illumination regime. Histograms of CFUs obtained through plating the initial timepoint of experiments shown in Figure 5B. The treatments were compared using ANOVA (p-value > 0.05).

A.

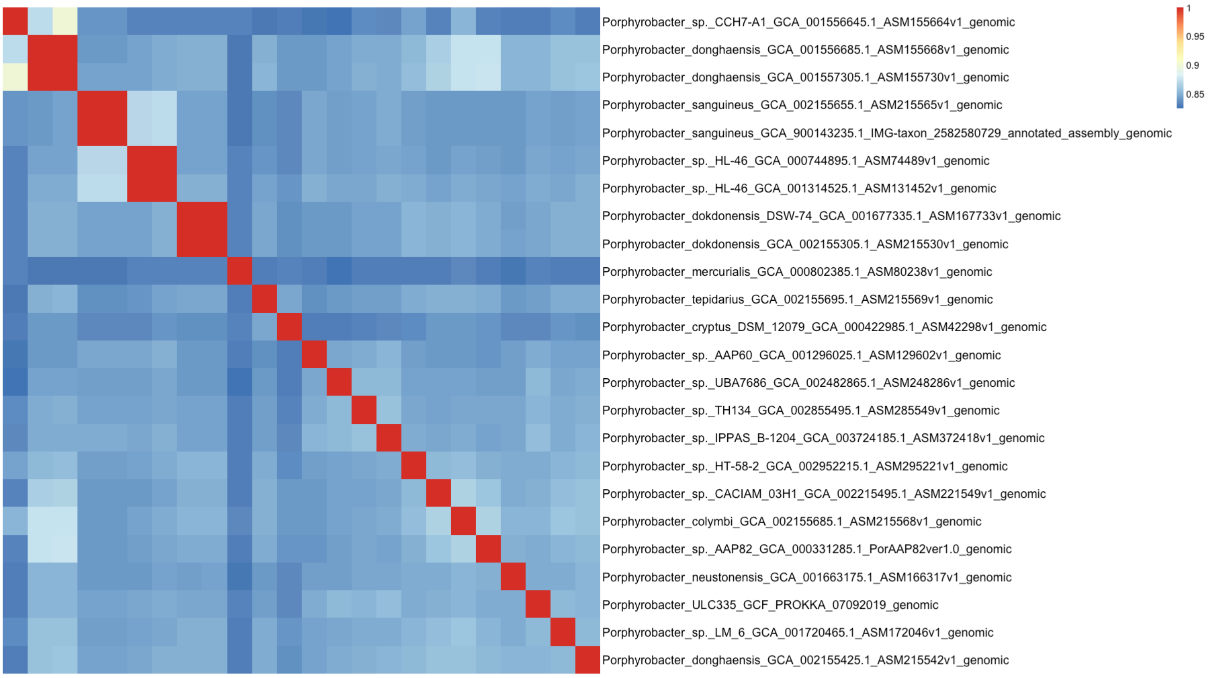

B.

Reference genome	DDH (%)
Porphyrobacter_sp._UBA7686_GCA_002482865.1_ASM248286v1	25.8
Porphyrobacter_sp._IPPAS_B-1204_GCA_003724185.1_ASM372418v1	25.6
Porphyrobacter_sp._CACIAM_03H1_GCA_002215495.1_ASM221549v1	25.3
Porphyrobacter_colymbi_GCA_002155685.1_ASM215568v1	25.1
Porphyrobacter_donghaensis_GCA_001557305.1_ASM155730v1	24.9
Porphyrobacter_sp._TH134_GCA_002855495.1_ASM285549v1	24.8
Porphyrobacter_donghaensis_GCA_002155425.1_ASM215542v1	24.8
Porphyrobacter_donghaensis_GCA_001556685.1_ASM155668v1	24.7
Porphyrobacter_sp._LM_6_GCA_001720465.1_ASM172046v1	24.5
Porphyrobacter_sp._AAP82_GCA_000331285.1_PorAAP82ver1.0	24.2
Porphyrobacter_sp._HT-58-2_GCA_002952215.1_ASM295221v1	23.9
Porphyrobacter_sp._HL-46_GCA_001314525.1_ASM131452v1	23.7
Porphyrobacter_sp._AAP60_GCA_001296025.1_ASM129602v1	23.2
Porphyrobacter_neustonensis_GCA_001663175.1_ASM166317v1	23.0
Porphyrobacter_tepidarius_GCA_002155695.1_ASM215569v1	22.6
Porphyrobacter_dokdonensis_GCA_002155305.1_ASM215530v1	22.5
Porphyrobacter_sanguineus_GCA_002155655.1_ASM215565v1	22.5
Porphyrobacter_sanguineus_GCA_900143235.1_IMG-taxon_2582580729_annotated_assembly	22.5
Porphyrobacter_dokdonensis_DSW-74_GCA_001677335.1_ASM167733v1	22.5
Porphyrobacter_sp._HL-46_GCA_000744895.1_ASM74489v1	22.4
Porphyrobacter_cryptus_DSM_12079_GCA_000422985.1_ASM42298v1	20.9
Porphyrobacter_mercurialis_GCA_000802385.1_ASM80238v1	18.6
Porphyrobacter_sp._CCH7-A1_GCA_001556645.1_ASM155664v1	18.5

C.

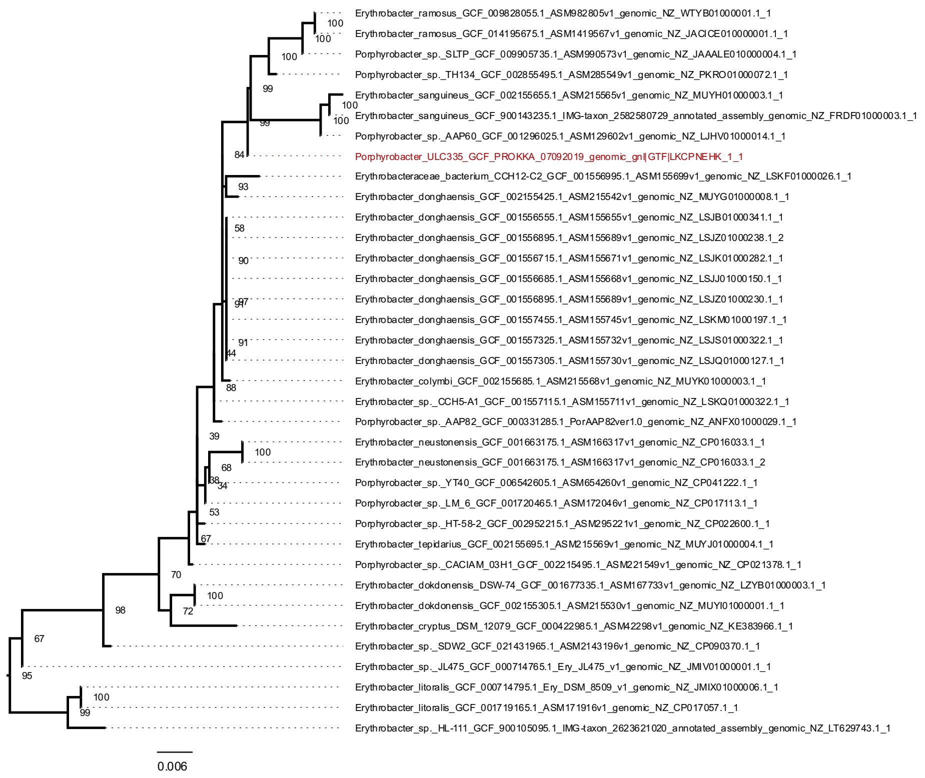

Erythrobacter_ramosus_GCF_009828055.1_ASM982805v1_genomic_NZ_WTYB01000001.1_1
Erythrobacter_ramosus_GCF_014195675.1_ASM1419567v1_genomic_NZ_JACICE010000001.1_1
Porphyrobacter_sp._SLTP_GCF_009905735.1_ASM990573v1_genomic_NZ_JAAALE010000004.1_1
Porphyrobacter_sp._TH134_GCF_002855495.1_ASM285549v1_genomic_NZ_PKRO01000072.1_1
Erythrobacter_sanguineus_GCF_002155655.1_ASM215565v1_genomic_NZ_MUYH01000003.1_1
Erythrobacter_sanguineus_GCF_900143235.1_IMG-taxon_2582580729_annotated_assembly_genomic_NZ_FRDF01000003.1_1
Porphyrobacter_sp._AAP60_GCF_001296025.1_ASM129602v1_genomic_NZ_LJHV01000014.1_1
Porphyrobacter_ULC335_GCF_PROKKA_07092019_genomic_gnl|GTF|LKCPNEHK_1_1
Erythrobacteraceae_bacterium_CCH12-C2_GCF_001556995.1_ASM155699v1_genomic_NZ_LSKF01000026.1_1
Erythrobacter_donghaensis_GCF_002155425.1_ASM215542v1_genomic_NZ_MUYG01000008.1_1
Erythrobacter_donghaensis_GCF_001556555.1_ASM155655v1_genomic_NZ_LSJB01000341.1_1
Erythrobacter_donghaensis_GCF_001556895.1_ASM155689v1_genomic_NZ_LSJZ01000238.1_2
Erythrobacter_donghaensis_GCF_001556715.1_ASM155671v1_genomic_NZ_LSJK01000282.1_1
Erythrobacter_donghaensis_GCF_001556685.1_ASM155668v1_genomic_NZ_LSJJ01000150.1_1
Erythrobacter_donghaensis_GCF_001556895.1_ASM155689v1_genomic_NZ_LSJZ01000230.1_1
Erythrobacter_donghaensis_GCF_001557455.1_ASM155745v1_genomic_NZ_LSKM01000197.1_1
Erythrobacter_donghaensis_GCF_001557325.1_ASM155732v1_genomic_NZ_LSJS01000322.1_1
Erythrobacter_donghaensis_GCF_001557305.1_ASM155730v1_genomic_NZ_LSJQ01000127.1_1
Erythrobacter_colymbi_GCF_002155685.1_ASM215568v1_genomic_NZ_MUYK01000003.1_1
Erythrobacter_sp._CCH5-A1_GCF_001557115.1_ASM155711v1_genomic_NZ_LSKQ01000322.1_1
Porphyrobacter_sp._AAP82_GCF_000331285.1_PorAAP82ver1.0_genomic_NZ_ANFX01000029.1_1
Erythrobacter_neustonensis_GCF_001663175.1_ASM166317v1_genomic_NZ_CP016033.1_1
Erythrobacter_neustonensis_GCF_001663175.1_ASM166317v1_genomic_NZ_CP016033.1_2
Porphyrobacter_sp._YT40_GCF_006542605.1_ASM654260v1_genomic_NZ_CP041222.1_1
Porphyrobacter_sp._LM_6_GCF_001720465.1_ASM172046v1_genomic_NZ_CP017113.1_1
Porphyrobacter_sp._HT-58-2_GCF_002952215.1_ASM295221v1_genomic_NZ_CP022600.1_1
Erythrobacter_tepidarius_GCF_002155695.1_ASM215569v1_genomic_NZ_MUYJ01000004.1_1
Porphyrobacter_sp._CACIAM_03H1_GCF_002215495.1_ASM221549v1_genomic_NZ_CP021378.1_1
Erythrobacter_dokdonensis_DSW-74_GCF_001677335.1_ASM167733v1_genomic_NZ_LZYB01000003.1_1
Erythrobacter_dokdonensis_GCF_002155305.1_ASM215530v1_genomic_NZ_MUYI01000001.1_1
Erythrobacter_cryptus_DSM_12079_GCF_000422985.1_ASM42298v1_genomic_NZ_KE383966.1_1
Erythrobacter_sp._SDW2_GCF_021431965.1_ASM2143196v1_genomic_NZ_CP090370.1_1
Erythrobacter_sp._JL475_GCF_000714765.1_Ery_JL475_v1_genomic_NZ_JMIV01000001.1_1
Erythrobacter_litoralis_GCF_000714795.1_Ery_DSM_8509_v1_genomic_NZ_JMIX01000006.1_1
Erythrobacter_litoralis_GCF_001719165.1_ASM171916v1_genomic_NZ_CP017057.1_1
Erythrobacter_sp._HL-111_GCF_900105095.1_IMG-taxon_2623621020_annotated_assembly_genomic_NZ_LT629743.1_1
0.006

D.

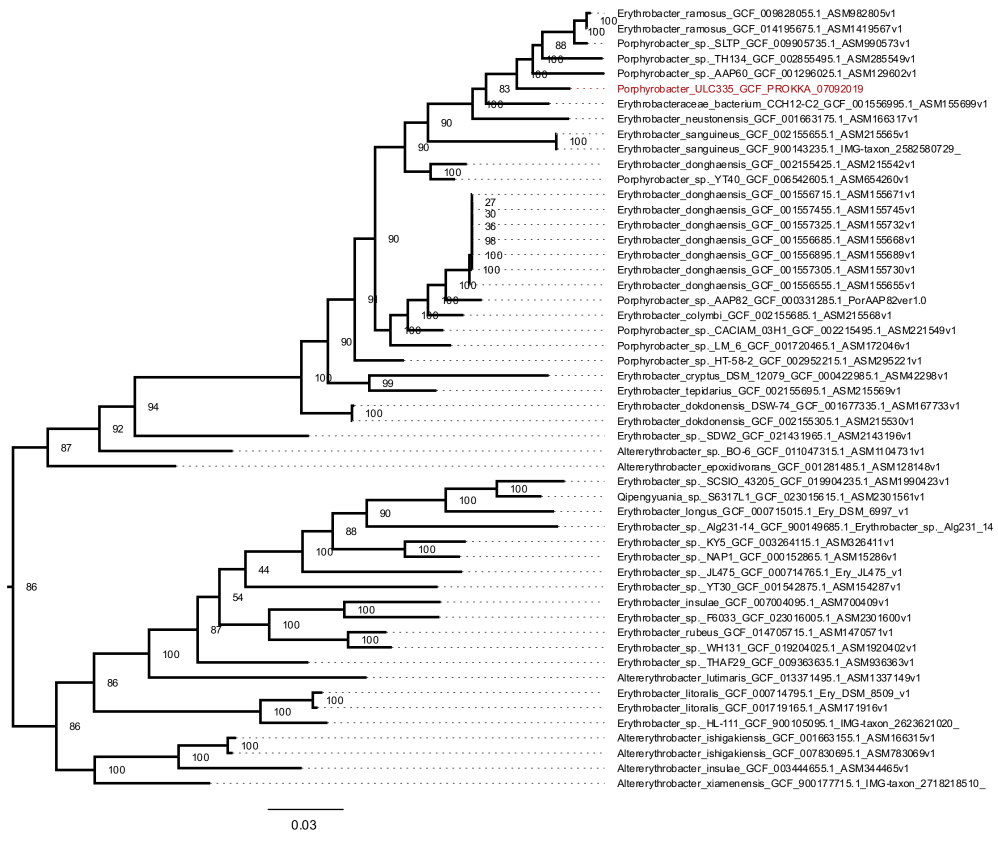

Erythrobacter_ramosus_GCF_009828055.1_ASM982805v1
Erythrobacter_ramosus_GCF_014195675.1_ASM1419567v1
Porphyrobacter_sp._SLTP_GCF_009905735.1_ASM990573v1
Porphyrobacter_sp._TH134_GCF_002855495.1_ASM285549v1
Porphyrobacter_sp._AAP60_GCF_001296025.1_ASM129602v1
Porphyrobacter_ULC335_GCF_PROKKA_07092019
Erythrobacteraceae_bacterium_CCH12-C2_GCF_001556995.1_ASM155699v1
Erythrobacter_neustonensis_GCF_001663175.1_ASM166317v1
Erythrobacter_sanguineus_GCF_002155655.1_ASM215565v1
Erythrobacter_sanguineus_GCF_900143235.1_IMG-taxon_2582580729_
Erythrobacter_donghaensis_GCF_002155425.1_ASM215542v1
Porphyrobacter_sp._YT40_GCF_006542605.1_ASM654260v1
Erythrobacter_donghaensis_GCF_001556715.1_ASM155671v1
Erythrobacter_donghaensis_GCF_001557455.1_ASM155745v1
Erythrobacter_donghaensis_GCF_001557325.1_ASM155732v1
Erythrobacter_donghaensis_GCF_001556685.1_ASM155668v1
Erythrobacter_donghaensis_GCF_001556895.1_ASM155689v1
Erythrobacter_donghaensis_GCF_001557305.1_ASM155730v1
Erythrobacter_donghaensis_GCF_001556555.1_ASM155655v1
Porphyrobacter_sp._AAP82_GCF_000331285.1_PorAAP82ver1.0
Erythrobacter_colymbi_GCF_002155685.1_ASM215568v1
Porphyrobacter_sp._CACIAM_03H1_GCF_002215495.1_ASM221549v1
Porphyrobacter_sp._LM_6_GCF_001720465.1_ASM172046v1
Porphyrobacter_sp._HT-58-2_GCF_002952215.1_ASM295221v1
Erythrobacter_cryptus_DSM_12079_GCF_000422985.1_ASM42298v1
Erythrobacter_tepidarius_GCF_002155695.1_ASM215569v1
Erythrobacter_dokdonensis_DSW-74_GCF_001677335.1_ASM167733v1
Erythrobacter_dokdonensis_GCF_002155305.1_ASM215530v1
Erythrobacter_sp._SDW2_GCF_021431965.1_ASM2143196v1
Altererythrobacter_sp._BO-6_GCF_011047315.1_ASM1104731v1
Altererythrobacter_epoxidivorans_GCF_001281485.1_ASM128148v1
Erythrobacter_sp._SCSIO_43205_GCF_019904235.1_ASM1990423v1
Qipengyuania_sp._S6317L1_GCF_023015615.1_ASM2301561v1
Erythrobacter_longus_GCF_000715015.1_Ery_DSM_6997_v1
Erythrobacter_sp._Alg231-14_GCF_900149685.1_Erythrobacter_sp._Alg231_14
Erythrobacter_sp._KY5_GCF_003264115.1_ASM326411v1
Erythrobacter_sp._NAP1_GCF_000152865.1_ASM15286v1
Erythrobacter_sp._JL475_GCF_000714765.1_Ery_JL475_v1
Erythrobacter_sp._YT30_GCF_001542875.1_ASM154287v1
Erythrobacter_insulae_GCF_007004095.1_ASM700409v1
Erythrobacter_sp._F6033_GCF_023016005.1_ASM2301600v1
Erythrobacter_rubeus_GCF_014705715.1_ASM1470571v1
Erythrobacter_sp._WH131_GCF_019204025.1_ASM1920402v1
Erythrobacter_sp._THAF29_GCF_009363635.1_ASM936363v1
Altererythrobacter_lutimaris_GCF_013371495.1_ASM1337149v1
Erythrobacter_litoralis_GCF_000714795.1_Ery_DSM_8509_v1
Erythrobacter_litoralis_GCF_001719165.1_ASM171916v1
Erythrobacter_sp._HL-111_GCF_900105095.1_IMG-taxon_2623621020_
Altererythrobacter_ishigakiensis_GCF_001663155.1_ASM166315v1
Altererythrobacter_ishigakiensis_GCF_007830695.1_ASM783069v1
Altererythrobacter_insulae_GCF_003444655.1_ASM344465v1
Altererythrobacter_xiamenensis_GCF_900177715.1_IMG-taxon_2718218510_
0.03

SFigure 24: Average nucleotide identity [ANI] and digital DNA-DNA hybridization [DDH] between the genomes of *Porphyrobacter* sp. ULC335 and several other *Porphyrobacter* strains representative of the diversity of the genus. A. ANI as calculated using PYANI [6]. The ANI of strain ULC335 is lower than 0.85 for a representative set of *Porphyrobacter* strains or species, which suggests that strain ULC335 may be a new *Porphyrobacter* species. B. The DDH was estimated using the digital Genome-to-Genome Distance Calculator algorithm recommended by the Leibniz Institute DSMZ (Formula 2) [7]. The DDH between *Porphyrobacter* sp. ULC335 and a representative set of *Porphyrobacter* strains or species is estimated below 30%, which suggests that strain ULC335 may be a new *Porphyrobacter* species. C. Phylogenetic tree of the closest relatives of strain ULC335 based on the full 16S rRNA gene sequence. D. Phylogenetic tree of the closest relatives of strain ULC335 based on 27 concatenated conserved proteins. Strain ULC335 is shown in red. *iqtree* bootstrap values are shown at the nodes (max. 100). Scale bars: average number of substitutions per site.

Supplementary Methods

RESSOURCE AVAILABILITY

Lead contact

Requests for information or strains should be directed to the lead contact, Diego Gonzalez (diego.gonzalez@unine.ch).

Materials availability

Porphyrobacter sp. ULC335 and its transposon mutants are available from the lead contact.

Data and code availability

The *Porphyrobacter* sp. ULC335 genome is publicly available on the NCBI repository (SAMN19986570). The RNA-sequencing data is publicly available on the NCBI Gene Expression Omnibus (GEO) repository (GSE245047).

EXPERIMENTAL MODEL AND SUBJECT DETAILS

Porphyrobacter sp. ULC335 was isolated from a BG11 Petri dish on which *Snowella* sp. ULC335 and its associated community of heterotrophs were plated. It formed isolated small orange to red colonies on the agar. After two initial reisolation steps on PYE [8], it was confirmed pure by microscopy and stocked at -80°C in 20% glycerol. Further cultivation was done on BG11-P (see below). The ULC335 community, the *Porphyrobacter* sp. ULC335 strain and its transposon mutants used in this article are listed and referenced in the table below.

Name	Description	Reference
ULC335 community	Microbial community (about 12 dominant heterotrophs) co-cultured with the cyanobacterium *Snowella* sp. ULC335.	[9]
Porphyrobacter sp. ULC335	Wild-type strain isolated from ULC335 on BG11 and propagated on BG11-P.	This study.
B^-C^+	*Porphyrobacter* sp. ULC335 mutant. Tn5 transposon inserted in *bchH* (at 3'319'193, KVF90_15790).	This study.
B^+C^-	*Porphyrobacter* sp. ULC335 mutant. Tn5 transposon inserted in *crtI* (at 1'406'641, KVF90_06770).	This study.
B^-C^-	*Porphyrobacter* sp. ULC335 mutant. Tn5 transposon inserted in *ispA* (at 2'364'745, KVF90_11145).	This study.
$B^{++}C^{++}$	*Porphyrobacter* sp. ULC335 mutant. Tn5 transposon inserted in *ppaA* (at 3'311'612, KVF90_15770).	This study.

METHOD DETAILS

Standard growth conditions

Porphyrobacter sp. ULC335 was grown in BG11 media [10] supplemented with 0.5g/L tryptone (Oxoid) and 0.25g/L yeast extract (Oxoid), as well as 1/100 dilution of vitamin solution (hereafter, BG11-P). Vitamin solution contained (mg/L): Biotin (4), Folic acid (4), Pyridoxine-HCl (20), Riboflavine (10), Thiamine-HCl $2H_2O$ (10), Nicotinamide (10), D-Ca-pantothenate (10), Vitamin B12 (0.2), p-Aminobenzoic acid (10). Standard batch growth took place at 30°C with agitation. Growth in the Photon Systems Instruments Multi-Cultivator MC1000 cultivating device took place at 28°C. Batch cultures for competition and survival experiments were done in 24-well plates at 28°C with agitation. *Escherichia coli* was grown in LB (tryptone, 10 g/L; yeast extract 5 g/L; NaCl, 5 g/L; tryptone and yeast extract from Oxoid) at 37°C. Solid media were supplemented with 1.5% technical agar.

Real-time optical density monitoring

Real-time monitoring of the optical density of *Porphyrobacter* sp. ULC335 cultures was done in a Multi-cultivator MC1000 (Photon Systems Instruments) under warm-white LEDs at 150 to 180µmol/s/cm^2 at 28°C with mixing ensured by bubbling (eight culture tubes in parallel). OD680 was measured every 12 minutes (six individual measures per timepoint) with one tube used as blank to correct for measurement fluctuations due to changes in the room temperature. Transitions in illumination regimes (square wave) are reported on the graphs presented for each experiment. Cells were grown in BG11-P from an initial optical density <0.001.

UV-visible-close IR spectra

SFigure 2. The cell pellets collected from 12 ml of 48-hour old liquid cultures of *P. neustonensis* DSM9434 and *Porphyrobacter* sp. ULC335 were extracted with 700µl of 7:2 acetone-methanol solvent for 5 minutes; after centrifugation, the absorbance of the supernatant was measured over the UV-visible-close IR spectrum in quartz cuvettes (1 cm path length) with a UV-visible spectrophotometer (Thermo Scientific Genesys 10s).

SFigure 11. For whole-cell spectra, cells were washed once with NaCl 0.9% and resuspended five-fold concentrated in NaCl 0.9%. 350 to 900 nm spectra were obtained from 200µl in a 96-well plate with a spectrophotometer (SpectraMax i3x, Molecular Devices).

Thin Layer Chromatography

A 50 ml aliquot of three day-old batch cultures of relevant strains were centrifuged; the pellet was resuspended in 20 ml of NaCl 0.9% and centrifuged again. The pellet was resuspended into 1 ml of 7:2 acetone-methanol and left in the dark for 5 minutes; the supernatant was collected by centrifugation. About 100µl of supernatant were spotted onto a Silica 60 F_{254} Thin Layer Chromatography (TLC) plate (Merk, art. 5554), previously dehydrated at 120°C for one hour, and left to dry. The TLC plate was migrated in 8:0.75:0.2:0.8:0.25 petroleum ether:hexane:isopropanol:acetone: methanol. Migration front and spots were used to calculate retardation factors for each pigment. The main red pigment was eluted into 7:2 acetone-methanol and its

UV-visible-close IR spectrum was measured in quartz cuvettes (1 cm path length) as stated above.

Transposon library

5ml of 48-hours old *Porphyrobacter* sp. ULC335 culture were pelleted, washed twice with BG11-P, and combined with an equivalent pellet of a conjugation-competent *E. coli* strain harbouring pRL27 [11]. The mixture of bacteria was spotted on a BG11-P plate and incubated at 30°C for 4 hours. The bacterial spot was then collected and spread on a total of twenty BG11-P supplemented with 50µg/ml kanamycin and 10µg/ml chloramphenicol (to counterselect *E. coli*). About 5'000 colonies were collected and stocked at -80°C.

A collection of mutants whose color differed from the wild type was isolated on BG11-P plates or BG11 plates supplemented with 2.25g/L Tryptone and 0.75g/L Yeast extract. The B^+C^- and B^-C^- strains were white; the B^-C^+ strain was orange to light red rather than dark red like the wild type; the $B^{++}C^{++}$ was darker than the wild type. The mutants were restreaked twice to make sure that they were pure. The insertion sites were identified using the method previously published [11]. Briefly, the genomic DNA of the mutants was extracted using an ammonium-acetated-based protein precipitation protocol followed by ethanol-precipitation [12]. 1 µg of purified DNA was digested using BamHI (1 hour, 37°C); after deactivation of the restriction enzyme, the DNA was autoligated overnight at 4°C using the T4 ligase (NEB). The ligation product was transformed into *E. coli* DH5a-λpir using the heat shock method . The plasmid was

extracted and the insert was sequenced using the tpnRL17–1 primer (Microsynth). The insertion sites were identified using *blast* [13].

pufM PCR

SFigure 1. The *pufM* PCR was carried out with pufM-F and pufM-R primers using GoTaq (M3001, Promega) on genomic DNA extracted using Quick-DNA Fungal/Bacterial Miniprep Kit (Zymo Research) from 500µl of overnight liquid culture in BG11-P. Annealing temperature was 55°C.

Genome sequencing and annotation

The genomic DNA was purified from 3ml of *Porphyrobacter* sp. ULC335 48-hour culture using the Qiagen Genomic-tip 20/g kit. Sequencing was performed using the Pacific Biosciences (PacBio Sequel). The sequencer generated 38'568 polymerase reads (mean length: 31,681) and 262'709 subreads (mean length: 4'389 bps); mean final coverage was 314. The genome was assembled using PacBio CCS 4.1.0 for the read correction step and Flye 2.6 [14] with a target size of 5MB, 1000bps minimal overlap between reads, and five final polishing steps for the assembly. In its final state, the genome comprises only one circular contig.
The functional classification of the genome content was done using the online blastKOALA service (https://www.kegg.jp/blastkoala/) with the genus *Porphyrobacter* as a reference. The two first levels of the KEGG classification were used to make SFigure 7.

RNA-sequencing

The wild type and B^-C^+ strains were cultured under 12h/12h dark-light alternance with constant oxygenation in the MC1000 cultivation device (warm white LEDs, 150 µmol/s/cm^2). After three days in stationary phase, samples were taken at four different time points from three different replicates. Sampling was done one hour before and one hour after the light-dark or dark-light transition: Early Day (ED) was taken one hour after dark-light transition, Late Day (LD) one hour before light-dark transition, Early Night (EN) one hour after light-dark transition, Late Night (LN) one hour before dark-light transition. RNA was extracted from 1 ml culture (OD650=0.3) using TRIzol (Invitrogen) followed by purification on columns. Briefly, cells were lysed in 1 ml TRIzol Reagent at 65°C for 10 minutes and kept at room temperature for 5 minutes; 200µl of chloroform were added and, after mixing by inversion, incubation for 3 minutes at room temperature and centrifugation (15 minutes, 12000 x g, 4° C), 500µl of the upper phase were mixed with an equal volume of 70% EtOH and loaded on a RNeasy mini-kit column (Qiagen). After an on-column DNase treatment (RNase-free DNase set, Qiagen), columns were washed and RNA eluted in 40µl of RNase-free H2O according to the manual instructions. RNA was quantified using Nanodrop2000 (ThermoFisher Scientific) and Qubit 2.0 fluorometer (Qubit RNA Broad Range Assay kit, ThermoFisher Scientific), checked for degradation on an agarose gel, and checked for possible DNA contamination by qPCR using SybrGreen (Rotor-Gene SYBR Green PCR kit, Qiagen) on a Rotor-Gene Q thermocycler (Qiagen). RNA-sequencing was carried out at Novogene on Illumina NovaSeq systems after

ribosomal depletion and in-house library construction. An average of 40 to 70 million 150 bps pair-end reads were obtained per sample.

Reads were trimmed and filtered using *fastP* [15], and then mapped on the *Porphyrobacter* sp. ULC335 genome using *bowtie2* [16]. The number of reads per coding sequence was counted using *featureCounts* [17]. Exploratory analysis and statistical testing were carried out using *R*. A first analysis, based on the *DESeq2* package [18] and workflow, was used to determine which genes were differentially expressed in the wild type and B^-C^+ strains at each of the four time points (pairwise comparisons for each timepoint, adjusted p-value < 0.01). A second analysis, based on the *ImpulseDE2* package [19], was used to determine which genes were temporally regulated across the four time points in either the wild type or the mutant strain (adjusted p-value < 0.01). To assign each temporally regulated gene to a specific temporal profile (cluster), we used *hclust* (Euclidian algorithm, 15 clusters) on the Z-scores of the *rlog*-transformed count data for all four time points. The *ggVennDiagram* [20] and *ggplot* [21] libraries were used to plot the summary figures.

Phylogenetic analysis

The proteomes of alphaproteobacterial genera containing at least four complete genomic sequences were downloaded from the NCBI repository and searched, using *blastp* [13] (e-value<10E-5), for homologs of fourteen proteins belonging to the anoxygenic phototrophy superoperon (BchH, BchD, BchB, BchY, BchZ, BchN, PpsR, BchC, BchX, BchI, BchL, BchO, BchM, BchF from *Roseobacter denitrificans* OCh114). For genomes containing the full set, the fourteen proteins were concatenated, the

sequences were aligned using *muscle* 3.8.31 [22] and a phylogenetic tree was constructed using *iqtree* 2.1.1 [23] with default parameters (evolution model chosen automatically). In parallel, the homologs of a set of conserved ribosomal proteins, elongation factors and gyrases (L2, L3, L4, L5, L6, L14, L15, L16, L17, L18, L22, L23, L24, L29, L30, L36, S3, S4, S5, S7, S8, S10, S11, S12, S13, S14, S17, S19, Tu, G, GyrA, and GyrB from *Pseudomonas aeruginosa* PAO1) were retrieved from the same proteomes and a phylogenetic tree was constructed following the same procedure. The Erythrobacteraceae ribosomal and superoperon trees were constructed in a similar way; however, since the genomes available on NCBI repository are not always complete, alignments had to be curated semi-automatically to avoid artefacts due to missing proteins. The 16S ribosomal RNA gene tree of the strains most closely related to *Porphyrobacter* sp. ULC335 was extracted from a tree comprising all Erythrobacteraceae plus *Caulobacter crescentus* NA1000 (outgroup); the 16S ribosomal RNA gene was extracted using *rnammer* 1.2 [24]; the nucleotide sequence alignment was done using *muscle* 3.8.31 [22] and a phylogenetic tree was constructed using *iqtree* 2.1.1 [23] with default parameters (evolution model chosen automatically) and 1000 boostrap. The DDH (digital DNA-DNA hybridization) was estimated using the digital Genome-to-Genome Distance Calculator algorithm recommended by the Leibniz Institute DSMZ (Formula 2) [7]. The ANI (average nucleotide identify) as calculated using PYANI [6].

SFigure 3. Strains were considered to encode the anoxygenic phototrophy superoperon if they gave positive *blastp* matches with at least 13 out of the 14 proteins listed above. The strains were assigned to a genus and class based on NCBI classification scheme.

Analysis of the ULC335 community

SFile 1. The trace sequences from the ULC335 metagenome (SRR6814902) were downloaded from NCBI repositories, trimmed using *fastp* [15] reassembled using *metaspades* with standard parameters [25], and resulting contigs were classified using the *CLASSIFIER* software (http://rdp.cme.msu.edu/classifier/classifier.jsp)[26].

Flow cytometry and fluorescence microscopy

Live-dead staining. Cells from 500µl of culture were resuspended in the same volume of NaCl 0.9% and incubated in the dark for 30 minutes after the addition of 1µl Invitrogen Baclite Live/Dead stain (mixture of equal volumes of components A and B). Cells were analyzed by flow cytometry and fluorescence microscopy. For the flow cytometry, cells were analyzed on a BD Accuri C6 Plus Flow Cytometer from a 1/100 dilution in NaCl 0.9%. Thresholds: 500 on FSC-H and 6000 on SSC-H. The zones corresponding to the dead, compromised, and live subpopulations were determined empirically based on the density distribution of live and 2.5% glutaraldehyde-fixed cells on a FL1 (green; excitation at 488nm, 533/30 emission filter) and FL3 (red; excitation at 488nm, 670LP emission filter) scatter plot. Visualization and quantification was done with R [27] using the *flowCore* [28] library after margin and doublet events removal using *PeacoQC* [29]. The same cells were analyzed using fluorescence microscopy (Leica DM4 B, Leica DFC7000 T camera, 100x objective : Leica 1.25 oil PH3 (506313)) with GFP and RFP settings; cells on phase-contrast, GFP, and

RFP images were counted with FIJI [30] using a customized semi-automated script; analysis and plotting was performed with R base functions. Only dead and live subpopulations could be distinguished with fluorescence microscopy; a high-GFP and high-RFP signal “compromised” subpopulation could not be reliably identified, presumably because of the different optical settings and detection limits of flow cytometry and fluorescence microscopy.

<u>DyeCycle Orange staining.</u> Cells from 500µl of culture were resuspended in the same volume of NaCl 0.9%, fixed with 2.5% glutaraldehyde for 15 minutes, and incubated in the dark for 30 minutes after the addition of 1µl Invitrogen DyeCycle Orange stain. Cells were analyzed by flow cytometry on a BD Accuri C6 Plus from a 1/100 dilution in NaCl 0.9%. Thresholds: 500 FSC and 6000 SSC. Subpopulation analysis was done using R with base functions. The coordinates of the highest density in the regions of the 1N and 2N peaks were determined from the density of a 1024-break histogram of the FL1 fluorescence using the *density* function. The distance between the 1N and the 2N coordinates was used to predict the theoretical position of 3N and 4N peaks. The best combination of underlying 1N, 2N, 3N, and 4N logistic density distributions explaining the empirical density distribution was determined by tuning the scale factors. The lowest density point between the 1N and the 2N peaks on the empirical density plot was used as a proxy for the “actively replicating” population, the assumption being that high replication rates would lead to a continuum between the 1N and 2N peaks and the minimum between the two could be used as a replication index.

Survival and competition experiments

Bacteriochlorophyll *a* content depending on the growth phase. Strains of interest were grown in BG11-P medium for approximately 60h at 28°C with a 170 rpm shaking, in dark conditions. Cultures were adjusted to OD650 = 0.001 in BG11-P medium and distributed in a 24-well plate: 12 wells per strain containing 1.3 ml of culture. Three identical plates were prepared for the three light conditions tested: continuous light, continuous dark and 12h/12h dark-light cycles. Plates were incubated at 28°C with shaking. After 18h, 24h, 48h, 72h and 96h of growth, the cultures of two wells per strains were collected on each plate and OD650 measured in a SpectraMax i3x plate reader (Molecular Devices). 900µl of culture were centrifuged and pellets were resuspended in 1 ml of a 0.9% NaCl solution. After centrifugation, pellets were resuspended in 250µl of methanol, centrifuged again and the clear supernatant was transferred in a new tube. Tubes were kept in the fridge and protected from light. 200µl of supernatant were loaded in a 96-well plate with flat bottom. Absorbance at 771 nm was measured to evaluate the production of bacteriochlorophyll *a* by the strains of interest in the different conditions. A full spectrum from 350 to 1000 nm (every 2 nm) was also recorded to evaluate the presence of other pigments. Measures were acquired in a SpectraMax i3x plate reader; samples were proceeded in two sets of 6 to avoid evaporation. The relative amounts of Bchl*a* and carotenoids (460 and 480 nm) were obtained by dividing the absorbances by the absorbance for the wild type DL condition (reference).

Survival depending on the genotype and the culture conditions. Late exponential phase cultures of the strains of interest (wild type, $B^{-}C^{+}$, $B^{++}C^{++}$) were diluted to OD650=0.001 in BG11-P and grown in 24-well plates (28°C, 170 rpm) under continuous light, continuous dark, or 12h/12h dark-light cycles in triplicates. At the

beginning of stationary phase (at 48h), cultures were homogenized by pipetting and 100µl of a 1.6*10^{-5} dilution of each replicate were plated on BG11-P; the colony forming units (CFUs) were calculated from the plate counts. After four additional days spent in stationary phase (at 144h), the procedure was repeated and CFUs were calculated again. The CFUs at 48h were used as a baseline and survival was calculated as the percent remaining cells at 144h.

Competition experiments. Late exponential phase cultures of the two mutant strains (B^-C^+ and $B^{++}C^{++}$) were mixed with the wild type strain in equal proportions (OD650=0.001) and grown in 24-well plates (28°C, 170 rpm) under continuous light, continuous dark, or 12h/12h dark-light cycles in triplicates. The dual cultures were plated at 48h and 144h like for the survival experiment, and the ratios of the mutant to the wild type were calculated for both timepoints based on CFUs.

Primers used in this study

Name	Sequence	Reference
pufM-F	TAC GGS AAC CTG TWC TAC	[31, 32]
pufM-R	CCA TSG TCC AGC GCC AGA A	[9]
tpnRL17–1	AAC AAG CCA GGG ATG TAA CG	[11]

QUANTIFICATION AND STATISTICAL ANALYSIS

Details on the quantification and statistical analyses performed can be found in the relevant subsections of the “METHOD DETAILS” and in the figure legends.

ADDITIONAL RESOURCES

None.

References

1. Takaichi S, Shimada K, Ishidsu J. Monocyclic cross-conjugated carotenal from an aerobic photosynthetic bacterium, Erythrobacter longus. *Phytochemistry* 1988; **27**: 3605–3609.
2. Yurkov V, Gad'on N, Drews G. The major part of polar carotenoids of the aerobic bacteria Roseococcus thiosulfatophilus RB3 and Erythromicrobium ramosum E5 is not bound to the bacteriochlorophyll a-complexes of the photosynthetic apparatus. *Arch Microbiol* 1993; **160**.
3. Koblížek M, Béjà O, Bidigare RR, Christensen S, Benitez-Nelson B, Vetriani C, et al. Isolation and characterization of Erythrobacter sp. strains from the upper ocean. *Arch Microbiol* 2003; **180**: 327–338.
4. Zarzycki J, Fuchs G. Coassimilation of Organic Substrates via the Autotrophic 3-Hydroxypropionate Bi-Cycle in Chloroflexus aurantiacus. *Appl Environ Microbiol* 2011; **77**: 6181–6188.
5. Selyanin V, Hauruseu D, Koblížek M. The variability of light-harvesting complexes in aerobic anoxygenic phototrophs. *Photosynth Res* 2016; **128**: 35–43.
6. Pritchard L, Glover RH, Humphris S, Elphinstone JG, Toth IK. Genomics and taxonomy in diagnostics for food security: soft-rotting enterobacterial plant pathogens. *Anal Methods* 2016; **8**: 12–24.
7. Meier-Kolthoff JP, Carbasse JS, Peinado-Olarte RL, Göker M. TYGS and LPSN: a database tandem for fast and reliable genome-based classification and nomenclature of prokaryotes. *Nucleic Acids Research* 2022; **50**: D801–D807.
8. Ely B. Genetics of Caulobacter crescentus. *Methods in Enzymology*. 1991. Elsevier, pp 372–384.
9. Cornet L, Bertrand AR, Hanikenne M, Javaux EJ, Wilmotte A, Baurain D. Metagenomic assembly of new (sub)polar Cyanobacteria and their associated microbiome from non-axenic cultures. *Microbial Genomics* 2018; **4**.
10. Andersen RA (ed). Algal culturing techniques. 2005. Elsevier/Academic Press, Burlington, Mass.
11. Larsen RA, Wilson MM, Guss AM, Metcalf WW. Genetic analysis of pigment biosynthesis in Xanthobacter autotrophicus Py2 using a new, highly efficient transposon mutagenesis system

that is functional in a wide variety of bacteria. *Arch Microbiol* 2002; **178**: 193–201.

12. Hereward J. DIY Gentra Puregene Protocol. *JAMES HEREWARD* .

13. Camacho C, Coulouris G, Avagyan V, Ma N, Papadopoulos J, Bealer K, et al. BLAST+: architecture and applications. *BMC Bioinformatics* 2009; **10**: 421.

14. Kolmogorov M, Bickhart DM, Behsaz B, Gurevich A, Rayko M, Shin SB, et al. metaFlye: scalable long-read metagenome assembly using repeat graphs. *Nat Methods* 2020; **17**: 1103–1110.

15. Chen S, Zhou Y, Chen Y, Gu J. fastp: an ultra-fast all-in-one FASTQ preprocessor. *Bioinformatics* 2018; **34**: i884–i890.

16. Langmead B, Salzberg SL. Fast gapped-read alignment with Bowtie 2. *Nat Methods* 2012; **9**: 357–359.

17. Liao Y, Smyth GK, Shi W. featureCounts: an efficient general purpose program for assigning sequence reads to genomic features. *Bioinformatics* 2014; **30**: 923–930.

18. Love MI, Huber W, Anders S. Moderated estimation of fold change and dispersion for RNA-seq data with DESeq2. *Genome Biol* 2014; **15**: 550.

19. Fischer DS. ImpulseDE2: Differential expression analysis of longitudinal count data sets. 2019.

20. Gao C-H. ggVennDiagram: A 'ggplot2' Implement of Venn Diagram. 2022.

21. Wickham H. ggplot2: Elegant Graphics for Data Analysis. 2016. Springer-Verlag New York.

22. Edgar RC. MUSCLE: multiple sequence alignment with high accuracy and high throughput. *Nucleic Acids Research* 2004; **32**: 1792–1797.

23. Nguyen L-T, Schmidt HA, von Haeseler A, Minh BQ. IQ-TREE: A Fast and Effective Stochastic Algorithm for Estimating Maximum-Likelihood Phylogenies. *Molecular Biology and Evolution* 2015; **32**: 268–274.

24. Lagesen K, Hallin P, Rødland EA, Stærfeldt H-H, Rognes T, Ussery DW. RNAmmer: consistent and rapid annotation of ribosomal RNA genes. *Nucleic Acids Research* 2007; **35**: 3100–3108.

25. Nurk S, Meleshko D, Korobeynikov A, Pevzner PA. metaSPAdes: a new versatile metagenomic assembler. *Genome Res* 2017; **27**: 824–834.

26. Wang Q, Garrity GM, Tiedje JM, Cole JR. Naïve Bayesian Classifier for Rapid Assignment of rRNA Sequences into the New Bacterial Taxonomy. *Appl Environ Microbiol* 2007; **73**: 5261–5267.

27. R Core Team. R: A Language and Environment for Statistical Computing. 2021. R Foundation for

Statistical Computing, Vienna, Austria.

28. Ellis B, Haaland P, Hahne F, Meur NL, Gopalakrishnan N, Spidlen J, et al. flowCore: flowCore: Basic structures for flow cytometry data. 2021.

29. Emmaneel A. PeacoQC: Peak-based selection of high quality cytometry data. 2021.

30. Schindelin J, Arganda-Carreras I, Frise E, Kaynig V, Longair M, Pietzsch T, et al. Fiji: an open-source platform for biological-image analysis. *Nat Methods* 2012; **9**: 676–682.

31. Achenbach LA, Carey J, Madigan MT. Photosynthetic and Phylogenetic Primers for Detection of Anoxygenic Phototrophs in Natural Environments. *Appl Environ Microbiol* 2001; **67**: 2922–2926.

32. Béjà O, Suzuki MT, Heidelberg JF, Nelson WC, Preston CM, Hamada T, et al. Unsuspected diversity among marine aerobic anoxygenic phototrophs. *Nature* 2002; **415**: 630–633.
